# The Streptomyces Metabolite Thiostrepton Inhibits Regulatory T Cell Differentiation and Function to Boost Antitumor Immune Responses

**DOI:** 10.1002/eji.70035

**Published:** 2025-08-17

**Authors:** Luana Silva, Luís Almeida, Fatima Al‐Naimi, Daniele Carvalho Nascimento, Aleksandra Lopez Krol, Luis Eduardo Alves Damasceno, Hakim Echchannaoui, José Carlos Alves‐Filho, Luciana Berod, Tim Sparwasser

**Affiliations:** ^1^ Institute of Medical Microbiology and Hygiene University Medical Center of the Johannes Gutenberg‐University Mainz Germany; ^2^ Department of Pharmacology Center for Research in Inflammatory Diseases Ribeirao Preto Medical School University of Sao Paulo Ribeirao Preto Brazil; ^3^ Department of Hematology Oncology, and Pneumology University Medical Center (UMC) and University Cancer Center (UCT) Johannes Gutenberg University Mainz Germany; ^4^ Research Center for Immunotherapy (FZI) University Medical Center of the Johannes Gutenberg University Mainz Mainz Germany; ^5^ Institute For Molecular Medicine University Medical Center of the Johannes Gutenberg‐University Mainz Germany

**Keywords:** immune responses, immunotherapy, regulatory T cells, T cells, tumor immunology

## Abstract

Regulatory T cells (Tregs) are associated with enhanced tumor progression and reduced therapy response rates. Therefore, overcoming the Treg‐mediated immunosuppressive barrier within the tumor to enhance antitumor immune responses is of central interest to advance cancer immunotherapy. To date, no tools exist that can be exploited to dampen Treg function and differentiation in vivo. Here, we show for the first time that the antibiotic thiostrepton exerts a potent inhibitory effect on Tregs. Mechanistically, thiostrepton disrupts Treg differentiation, reduces the expression of Treg activation markers, and inhibits Treg suppressive functions. Accordingly, using an MC38 tumor model, we demonstrate that thiostrepton treatment reduces the number of intratumoral Foxp3^+^ Treg cells and prevents tumor growth. These effects are conserved in human T cells, as thiostrepton also inhibits the differentiation of human Tregs. Our findings highlight thiostrepton as a promising Treg‐targeting immunomodulatory compound with the potential to enhance antitumor immune responses.

## Graphical abstract

Regulatory T cells (Tregs) promote tumor progression and therapy resistance by suppressing antitumor immunity. Here, we identify the antibiotic thiostrepton as a novel Treg‐targeting immunomodulator for cancer immunotherapy. Thiostrepton impairs Treg differentiation and suppressive capacity. In an MC38 model, thiostrepton treatment reduced intratumoral Foxp3⁺ Tregs and significantly inhibited tumor growth.

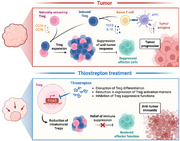

## 1 Introduction

Over the past decade, we have witnessed remarkable progress in our understanding of cancers—highly heterogeneous diseases characterized by a broad spectrum of hallmark pathological features and responsible for millions of deaths each year. Tumors represent complex, dynamic systems shaped by genetic and epigenetic changes, and, akin to healthy tissues, they form complex ecosystems that adapt to support survival and progression. The tumor microenvironment (TME) is a sophisticated structure comprising several noncancerous cell types, such as fibroblasts, endothelial cells, stromal cells, and immune cells, among others, embedded in a remodeled extracellular matrix (ECM) [1, 2, 3]. Interactions between the tumor and the TME actively regulate key processes such as tumor progression, immune evasion, angiogenesis, and metastasis formation [4].

T lymphocytes are key immune components of the TME, which engage in a complex relationship with cancer cells. On one hand, effector T lymphocytes, especially CD8^+^ cytotoxic T cells (CTLs), partake in direct killing of the tumor cells [5, 6], while type 1 helper (T_H_1) CD4^+^ T cells assist in the recruitment and effectiveness of CTLs [7, 8]. Conversely, regulatory T cells (Tregs) are specifically recruited to or generated within the TME to dampen antitumor immunity [9, 10]. Currently, it is widely accepted that Tregs promote immune evasion and tumor growth by overcoming the effector functions of effector T lymphocytes. Indeed, an increased Treg to CTL ratio has been directly correlated with poor prognosis and reduced patient survival in different types of malignancies [11].

Advances in our understanding of the critical elements within the TME and their crosstalk with immune cells are critical in fostering the development of innovative therapeutic approaches. Current FDA‐approved immunotherapies include antigen‐presenting cells (APC) vaccination strategies [12, 13], immune checkpoint inhibition (ICI) [14], CAR‐T cell therapy, and cancer vaccines [15, 16].

Although these approaches have transformed cancer treatment, especially in hematologic malignancies, many solid tumors remain resistant. This can be attributed to a number of factors, such as tumor heterogeneity, epigenetic modifications, off‐target adverse effects, immune escape mechanisms, and, especially, a highly immunosuppressive TME [17, 18, 19]. Therefore, there is an urgent need to find alternative and complementary therapeutic strategies for cancer immunotherapy.

As described above, Treg cells are typically abundant in the TME, contributing to ineffective antitumor immune responses. These cells are characterized by the expression of the transcription factor Forkhead box protein P3 (Foxp3) [20] and are responsible for maintaining peripheral immune tolerance through their suppressive effect on effector T cells and APCs [21]. Prior studies, including ours, have shown that the selective depletion of Tregs enhances immune‐mediated tumor control in mice [22, 23, 24, 25, 26, 27, 28, 29], making them a primary target within the TME for novel immunotherapies. Although there are currently no FDA‐approved therapies developed specifically to target intratumoral Treg cells, several strategies are under investigation in preclinical studies that aim to exploit unique immunological and metabolic features of tumor‐associated Tregs, such as CD36 expression [30, 31]. Furthermore, certain clinically approved immunotherapies, including cytotoxic T‐lymphocyte–associated protein 4 (CTLA‐4)–blocking antibodies such as ipilimumab, have been suggested to influence Treg biology in the TME. However, clinical evidence regarding the impact of anti‐CTLA‑4 therapy on intratumoral Tregs remains inconclusive—while some clinical studies suggest that these therapies may contribute to antitumor immunity in patients partly by modulating or depleting Tregs, others report minimal or no significant changes in Treg frequencies following treatment [32, 33, 34]. These emerging approaches underscore the therapeutic potential of targeting Tregs, despite the current lack of agents that selectively and exclusively target this cell population in the clinic.

Thiostrepton is a natural thiopeptide antibiotic produced by *Streptomyces* strains ubiquitously found in soil and water. This metabolite functions as a protein translation inhibitor and is already in use in veterinary medicine for the treatment of infections caused by Gram‐positive bacteria [35, 36]. It has also been postulated to have tumoricidal activity, mostly related to its chemical inhibition of oncogenic transcription factor Forkhead box M1 (FOXM1) in tumor cells [37, 38]. However, its effects on the immune system, particularly on Treg cells, have not been characterized.

In the present study, we show that thiostrepton serves as a potent inhibitor of Treg differentiation and function in both murine and human systems. in vivo, thiostrepton treatment decreased intratumoral Treg abundance and significantly reduced tumor burden in a murine model of subcutaneous MC38 tumor. Together, these findings warrant further investigation into the therapeutic potential of thiostrepton to “release the brakes” exerted by Tregs on antitumor immunity.

## 2 Results

### 2.1 Thiostrepton Promotes Efficient Antitumor Immunity and Selectively Reduces Treg Cells Within the TME

Thiostrepton has been mostly associated with exerting antitumor activity in various cancer models [39, 40, 41, 42, 43, 44]. However, whether thiostrepton elicits a direct impact on regulatory T cells in the context of tumors has not yet been characterized.

**FIGURE 1:**
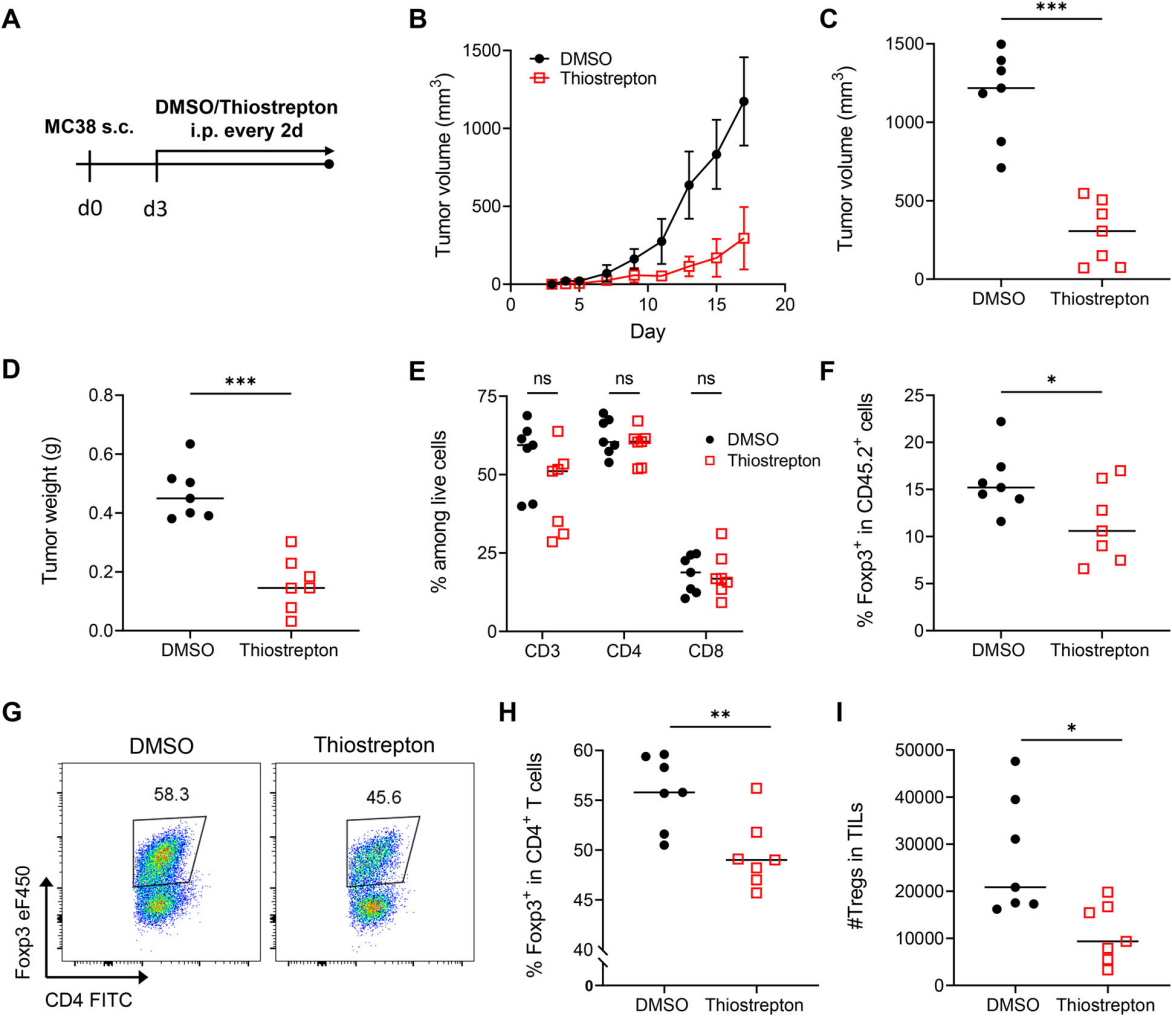
**Thiostrepton promotes efficient antitumor immunity and selectively reduces Treg cells within the TME**. C57BL/6 mice were subcutaneously injected with 4 × 10^5^ MC38 cells and subsequently treated with thiostrepton or vehicle (DMSO) every other day, beginning on day 3 posttumor inoculation. **(A)** Schematic overview of the experimental layout. **(B)** Representative graph depicting tumor growth in individual DMSO‐ and thiostrepton‐treated mice. **(C)** Tumor volume in mm^3^ and **(D)** weight on day 17 (last day) of the experiment. **(E–I)** Tumor‐infiltrating lymphocytes were isolated and analyzed by flow cytometry. **(E)** Frequencies of CD3^+^, CD4^+^, and CD8^+^ T cells among live cells. **(F)** Frequency of Tregs within CD45.2^+^ cells. **(G)** Representative contour plots and **(H)** frequency of Tregs within CD4^+^ T cells. **(I)** Absolute number of infiltrating Tregs in the tumor. The experiment was performed using *N* = 7 mice per treatment group (DMSO/thiostrepton). The Multiple unpaired *t*‐test was used for statistical analysis. Mean ± SD, *p < 0.05, **p < 0.01, ***p < 0.001, ns, not significant.

To assess this, we analyzed the effects of thiostrepton using the MC38 model. C57BL/6 mice were subcutaneously inoculated with 4 × 10^5^ MC38 cells and treated every two days with thiostrepton or vehicle (DMSO), starting on the third day after tumor inoculation (Figure 1A). The gating strategy is shown in Figure S1A–C. Thiostrepton treatment resulted in a significant reduction in tumor growth over time and a notable decrease in final tumor weight compared with vehicle‐treated controls (Figure 1B–D).

We next analyzed the cellular composition of the T cell compartment within the TME on day 17 after MC38 inoculation. Mice treated with thiostrepton exhibited comparable percentages of tumor‐infiltrating lymphocytes (TIL—CD3, CD4, or CD8) (Figure 1E). We further assessed the cytokine‐producing capacity of the TIL subsets and found no statistically significant differences (Figure S1D–F). However, the percentages of Foxp3⁺ Treg cells (Figure 1F–H), as well as absolute numbers (Figure 1I), were significantly reduced in thiostrepton‐treated mice, suggesting a hitherto undetected specific effect of thiostrepton on Treg cells. To evaluate the systemic effects of thiostrepton treatment, we examined changes in T cell populations and cytokine production within secondary lymphoid organs, including the spleen and draining lymph nodes (dLN). Treatment of mice with thiostrepton did not result in alterations in the cellular composition of the T cell compartment (Figure S1G,H), nor in cytokine production (Figure S1I).

These data suggest that thiostrepton modulates the tumor microenvironment by selectively reducing intratumoral Treg cells, contributing to improved antitumor immunity.

## 3 Thiostrepton Does Not Directly Affect Tumor Cell Growth nor Early T Cell Activation

**FIGURE 2:**
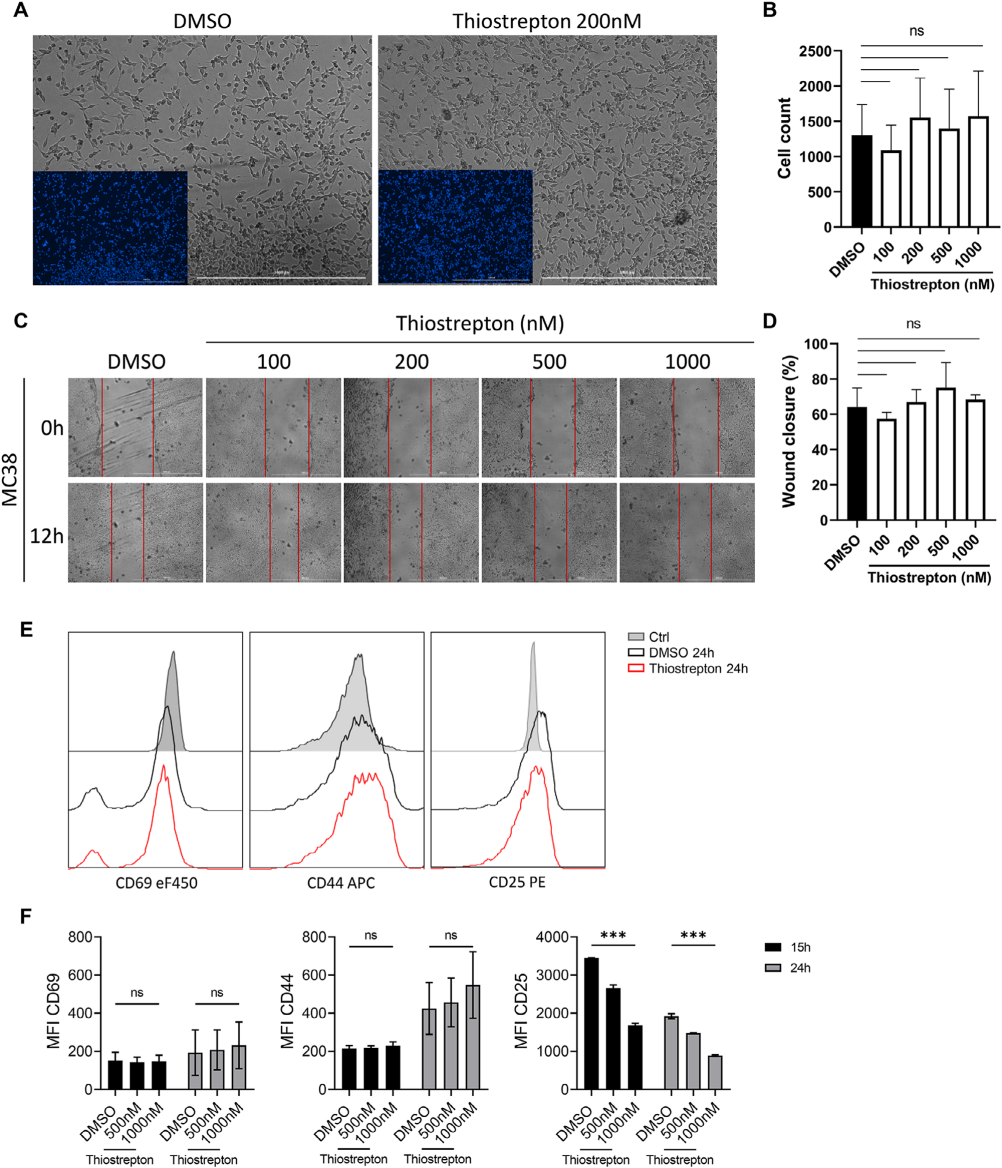
**Thiostrepton does not directly affect tumor cell growth nor early T cell activation. (A–D)** MC38 cells were treated with vehicle or increasing concentrations of thiostrepton. **(A)** Representative brightfield images of MC38 cells at 48 h posttreatment with DMSO/thiostrepton (4× magnification). Inset shows fluorescently labeled nuclei (Hoechst 33342). Scale bar (white) = 1000 µm. **(B)** Automated quantification of cell numbers in selected well sections. **(C)** Representative brightfield images of MC38 cells treated with DMSO/thiostrepton (4× magnification). Cells were seeded to 80% confluence in a monolayer, and a wound was created by firmly scratching with a 2 µL pipette tip. Scale bar (white) = 1000 µm. **(D)** Quantification of the percentages of wound‐healed areas 12 h after scratch. The relative distances between the edges of the wounds were measured at three different points. **(E, F)** Naïve T cells were cultured under iTreg‐polarizing conditions, in the presence of DMSO or thiostrepton for 15 and 24 h. **(E)** Representative histogram and **(F)** mean fluorescence intensity (MFI) of CD69, CD44, and CD25 expression. The gating strategy is shown in Figure S2A. Values are expressed as the mean ± SD, and a one‐way ANOVA test was used for statistical analysis. Mean ± SD, *p < 0.05, **p < 0.01, ***p < 0.001, ns, not significant. **(A‐D)** *N* = 2, *n* = 3; **(E, F)** *N* = 2, *n* = 3.

Since thiostrepton has been shown to directly inhibit the growth of and exert cytotoxic effects on various cancer cells [39, 41, 44], we wanted to understand whether the antitumor effects that we observed in vivo could be attributed to a direct cytotoxic effect on MC38 cells. Across the range of concentrations tested, thiostrepton did not significantly alter the number of MC38 cells after 2 days of culture (Figure 2A,B), suggesting that thiostrepton does not exert a direct, pronounced cytostatic or cytotoxic effect. To verify whether thiostrepton would repress cell growth and migration, we performed a wound‐healing assay on MC38 cells in the presence of increasing doses of thiostrepton (Figure 2C). The percentage of wound closure was calculated by the average decrease in distance measured between the edges of the wounds at least at three different points (Figure 2D). The wound‐healing assays revealed that MC38 cells treated with vehicle and thiostrepton healed effectively, with wounds decreasing to a similar degree after 12 h.

Next, we sought to evaluate whether the immunomodulatory effects of thiostrepton were present during early T cell activation and could affect viability. Naïve T cells were activated in vitro and treated with thiostrepton or vehicle for 15 and 24 h. The expression of early activation markers CD69, CD44, and CD25 [45, 46, 47] was then measured. We could observe that thiostrepton‐treated T cells exhibited comparable mean fluorescence intensity (MFI) for CD69, CD44, and lower MFI for CD25 compared with controls (Figure 2E,F), indicating that thiostrepton could be selectively impairing IL‐2 signaling, without blocking TCR engagement or the immediate early T cell activation response.

We then examined whether thiostrepton influences T cell homeostasis in vivo. Mice were administered thiostrepton or vehicle intraperitoneally every day for 7 days (Figure S3A). Flow cytometry analysis of lymph nodes and spleens (gating strategy shown in Figure S3B) revealed no significant differences in either the frequencies or absolute numbers of CD4⁺ and CD8⁺ T cells after thiostrepton treatment (Figure S3C,D). Additionally, the frequencies of central memory (CD62L^+^CD44^+^), effector (CD62L^−^CD44^+^), and naïve (CD62L^+^CD44^−^) CD4^+^ and CD8^+^ T cells were comparable between DMSO and thiostrepton‐treated mice (Figure S3E,F). Together, these results indicate that thiostrepton does not directly impair tumor cell growth nor the T cell compartment in homeostasis.

## 4 Thiostrepton Disrupts Treg Differentiation and Function in vitro

**FIGURE 3:**
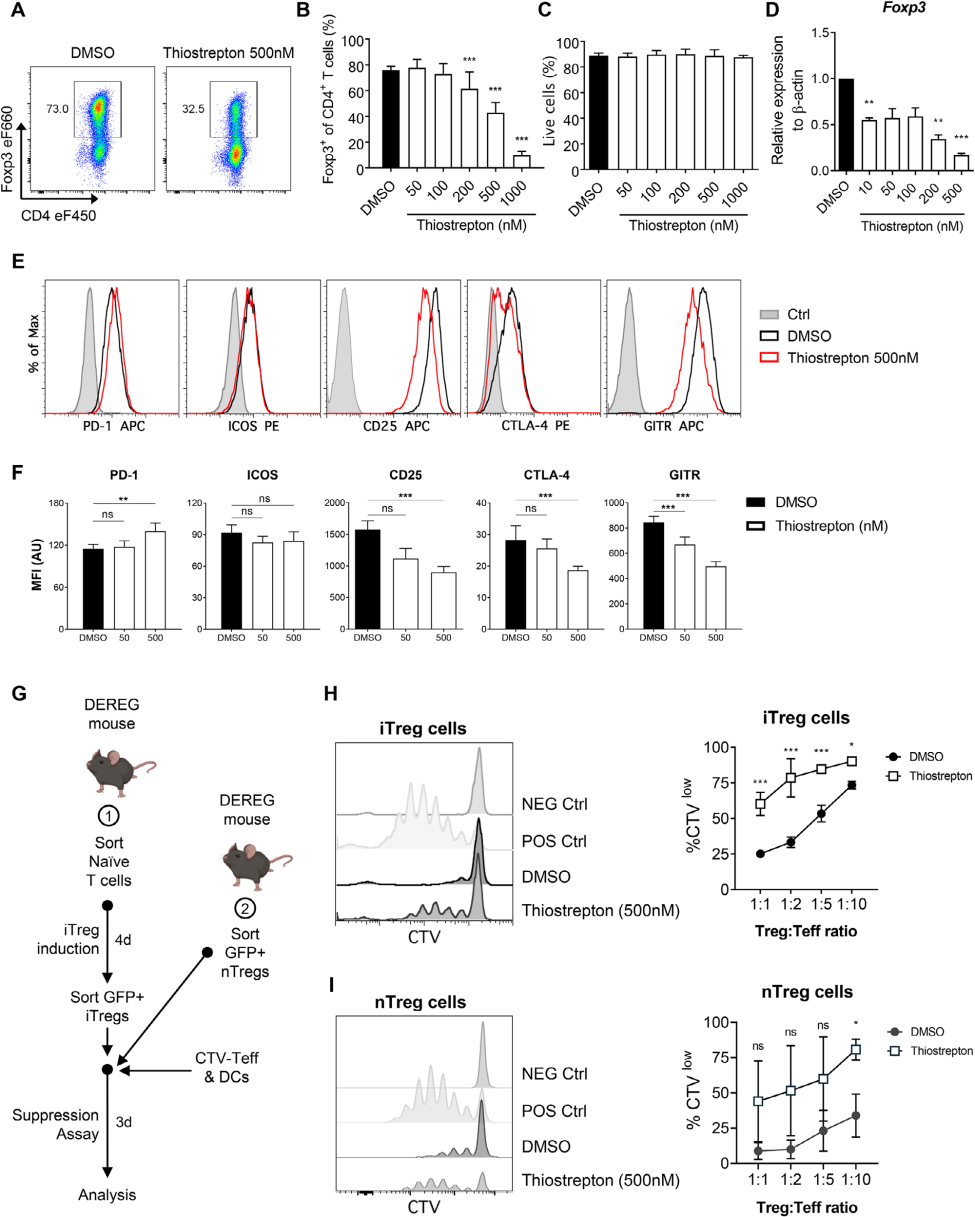
**Thiostrepton disrupts Treg differentiation and function in vitro. (A–F)** Naïve T cells polarized under iTreg‐inducing conditions were cultured in the presence of DMSO or increasing concentrations of thiostrepton. **(A)** Representative contour plots, **(B)** frequency of Foxp3^+^ iTregs and **(C)** viable cell counts on day 4 of culture. The gating strategy is shown in Figure S4A. **(D)** Foxp3 mRNA expression analyzed by qPCR on day 4 of culture. **(E)** Representative histograms and **(F)** median fluorescence intensity (MFI) of Treg‐associated markers in iTregs after vehicle or thiostrepton treatment. **(G–I)** The suppressive capacity of Tregs was tested by performing in vitro suppression assays. Effector T cell proliferation was measured according to the CellTrace Violet (CTV) fluorescence intensity across different Treg:Teff ratios. CTV^low^ Teff cells were considered to have proliferated. **(G)** Schematic representation of the assay: in 1, naïve T cells were polarized under iTreg conditions in the presence or absence of thiostrepton (200 nM), and sorted at d4 of culture based on GFP expression to use in the assay, data shown in **(H)**; in 2, sorted nTregs were sorted based on GFP expression and incubated with thiostrepton (200 nM) for 24 h prior to being used in the assay, data shown in (**I**). Representative (left) histograms and (right) %CTV^low^ cells depicting the proliferation of Teff cells in the presence of DMSO‐ or thiostrepton‐pretreated iTregs **(H)** and nTregs **(I)**. The corresponding gating strategy is shown in Figure S4B. Values are expressed as the mean ± SD, and asterisk values are significant compared with the control group by two‐way ANOVA, *p < 0.05, **p < 0.01, ***p < 0.001, ns, not significant. **(A–C)** *N* = 7, *n* = 3. **(D)** *N* = 2, *n* = 3. **(E, F)** *N* = 2, *n* = 3. **(H)** *N* = 3, *n* = 3. **(I)** *N* = 5, *n* = 3.

Our results indicate that thiostrepton treatment in vivo works primarily by directly targeting intratumoral Tregs (Figure 1F–H). Since Tregs are known to differentiate within the TME and suppress local immune responses [48, 49], we hypothesized that thiostrepton could directly inhibit Treg differentiation or function. To test this, we cultured naïve CD4^+^ T cells under iTreg‐polarizing conditions in the presence of increasing concentrations of thiostrepton. After four days, we observed a dose‐dependent decrease in Foxp3‐expressing cells in the presence of thiostrepton, with an estimated EC_50_ of around 500 nM (Figure 3A,B), without affecting cell viability (Figure 3C). This reduction was also evident at the transcriptional level, with *Foxp3* transcript levels displaying a similar dose‐dependent response (Figure 3D).

To further explore the kinetics of thiostrepton‐mediated inhibition of Treg differentiation, we measured the expression of Foxp3 during iTreg differentiation over time (Figure S5). Throughout the 4‐day culture period, the proportion of Foxp3^+^ cells remained lower in thiostrepton‐treated conditions, with ∼50% of cells expressing Foxp3 in the thiostrepton group (250 nM and 500 nM) versus ∼80% in vehicle‐treated cells at the end of the 4‐day cultures (Figure S5A,B), without compromising cell viability (Figure S5C).

Since Foxp3 upregulation and sustained expression are essential for Treg suppressive function [50], we next asked whether thiostrepton‐treated iTregs were functionally impaired. To explore this, we examined the expression of Treg‐associated activation and functional markers PD‐1, ICOS, CD25, CTLA‐4, and GITR [51, 52, 53, 54]. After 4 days of differentiation, thiostrepton‐treated iTregs displayed significantly decreased surface expression of CD25, CTLA‐4, and GITR compared with the respective controls. In contrast, the levels of PD‐1 and ICOS remained increased or unchanged, respectively, relative to baseline (Figure 3E,F). To assess the functional implications of these findings, we measured the suppressive capacity of thiostrepton‐treated Tregs. We performed in vitro suppression assays comparing thiostrepton‐treated iTregs with control‐treated iTregs, as well as nTregs (Figure 3G). We found that both thiostrepton‐treated iTregs and nTregs displayed a significantly lower suppressive capacity compared with controls (Figure 3H,I). Overall, these results show that thiostrepton is not only a potent inhibitor of Foxp3 induction and, consequently, Treg differentiation, but also impairs the suppressive capacity of differentiating and mature Tregs. This further supports its potential as a Treg‐targeted immunomodulatory agent.

## 5 Inhibition of Treg Cell Differentiation and Function by Thiostrepton Occurs via a Proliferation‐Independent Mechanism

**FIGURE 4:**
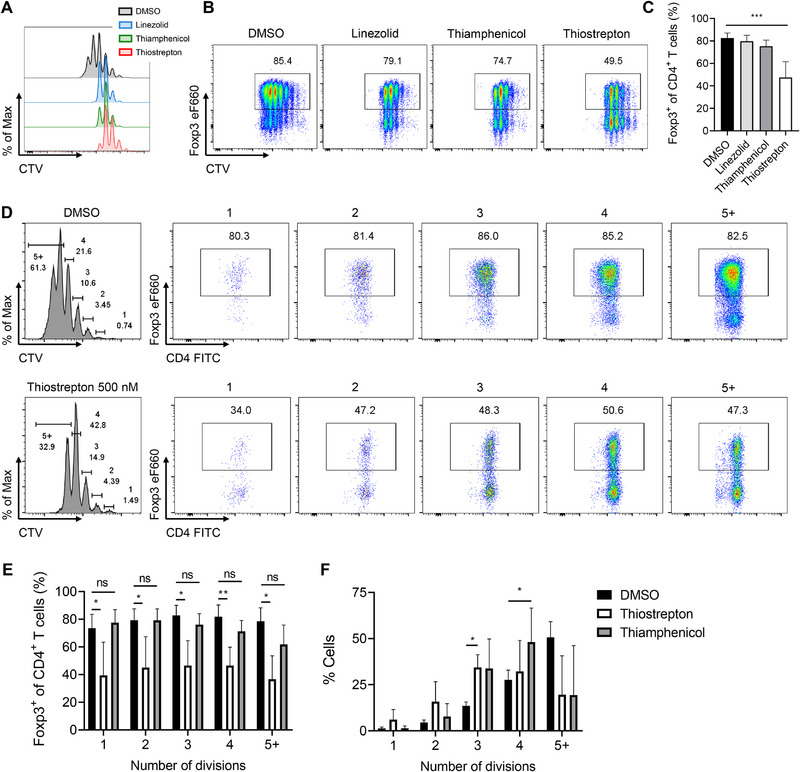
**Inhibition of Treg cell differentiation and function by thiostrepton occurs via a proliferation‐independent mechanism**. Naïve T cells were labeled with CellTrace Violet (CTV) and cultured under iTreg‐polarizing conditions in the presence of DMSO, linezolid (100 µM), thiamphenicol (100 µM), or thiostrepton (500 nM). **(** **A** **)** Representative histogram juxtaposing the proliferation of cells treated with linezolid (100 µM), thiamphenicol (100 µM), or thiostrepton (500 nM) with that of those treated with vehicle (DMSO), and **(** **B** **)** Representative histograms demonstrating the expression of Foxp3 versus proliferation according to each drug treatment. **(** **C** **)** Frequency of Foxp3^+^ iTregs across the different antibiotic treatments. **(** **D** **)** (Right) Representative histogram depicting different proliferation cycles of iTregs in the presence of DMSO or thiostrepton (500 nM) and (left) Representative histograms of Foxp3 frequencies across each proliferation cycle in the aforementioned conditions. Representative gating strategy is shown in Figure S6A. **(** **E** **)** Respective percentage of Foxp3^+^ cells across each individual proliferation cycle and **(** **F** **)** Pooled frequency of cells that have divided one, two, three, four, and five or more times, as indicated, in the presence of DMSO, thiamphenicol (100 µM), or thiostrepton (500 nM). Values are expressed as the mean ± SD, and asterisk values are significant compared with the control group by two‐way ANOVA, *p < 0.05, **p < 0.01, ***p < 0.001, ns, not significant. **(** **A–F** **)** *N* = 4, *n* = 2–3.

Having phenotypically characterized the inhibitory effect of thiostrepton on Tregs, we next aimed to elucidate the molecular mechanism behind it. Thiostrepton is a pleiotropic antibiotic with known antiproliferative properties [39, 42, 55]. To determine whether its inhibitory effect on Foxp3 expression in Tregs was merely a consequence of reduced proliferation, we compared its activity with that of linezolid and thiamphenicol—two antibiotics known to share thiostrepton's antiproliferative effect on multiple cell types [56, 57]. Under iTreg‐polarizing conditions, all three compounds similarly reduced the proliferation of iTreg cells compared with DMSO (Figure 4A,B). However, only thiostrepton significantly suppressed Foxp3 expression (Figure 4C). Furthermore, thiostrepton treatment led to decreased Foxp3 expression across all cell division cycles (Figure 4D–E), in contrast to thiamphenicol (Figure 4E), even though both antibiotics impacted proliferation to a similar extent (Figure 4F). Our results demonstrate that thiostrepton's inhibition of Treg cell differentiation is not directly caused by its effects on cell proliferation and, rather, takes place independently of it.

## 6 The Impairment in iTreg Differentiation Caused by Thiostrepton Treatment Is Not Attributable to Mitochondrial Dysfunction

**FIGURE 5:**
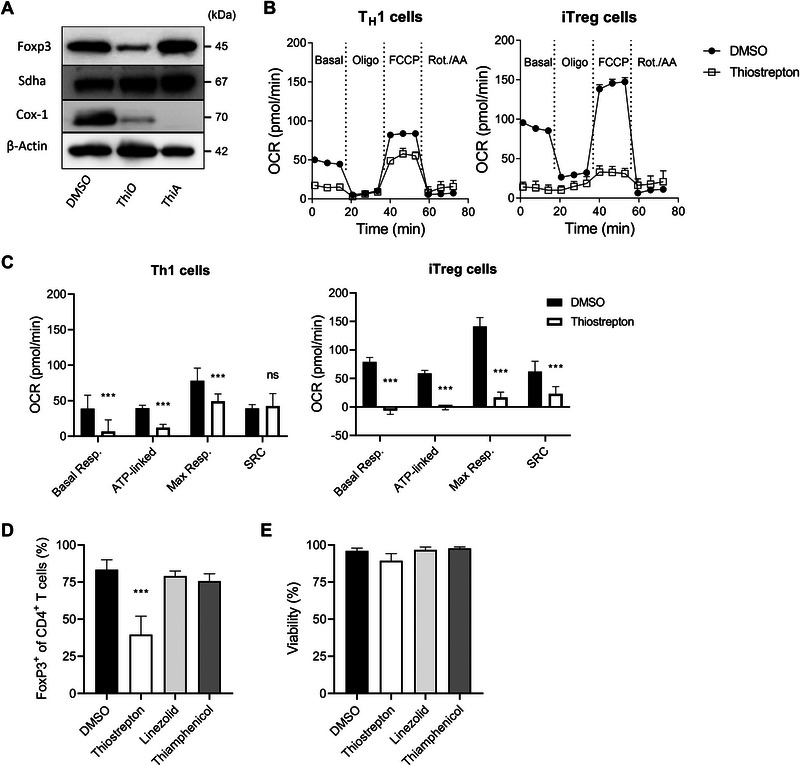
**The impairment in iTreg differentiation caused by thiostrepton treatment is not attributable to mitochondrial dysfunction**. **(** **A–C** **)** Mouse naïve T cells were cultured in iTreg‐polarizing conditions in the presence of DMSO, linezolid (100 µM), thiamphenicol (100 µM), or thiostrepton (500 nM). **(** **A** **)** Foxp3, Cox‐1, Sdha, and β‐Actin protein levels assessed by western blot as a readout of mitochondrial translation. Corresponding raw data in Figure S7A. **(** **B** **)** Representative graphs depicting the oxygen consumption rate (OCR) of in vitro differentiated T_H_1 cells (left) and iTregs (right) on day 4 of culture, treated with DMSO or thiostrepton (500 nM). **(** **C** **)** Quantification of basal respiration, ATP production, maximum respiration, and SRC in the aforementioned cultures. **(** **D** **)** Flow cytometry analysis of Foxp3 Treg cells frequency within CD4^+^ cells. **(** **E** **)** Assessment of cell viability under each condition. Values are expressed as the mean ± SD, and asterisk values are significant compared with the control group by one‐way ANOVA test (*p < 0.05). **(A)** *N* = 3, *n* = 2–3. **(** **B**, **C** **)** *N* = 3, *n* = 10. **(** **D**, **E** **)** *N* = 3, *n* = 2–3.

Mitochondrial metabolism is now recognized as an important key factor in T cell differentiation and function [58]. Thiostrepton is known to be a potent inhibitor of both the prokaryotic ribosome and the mitochondrial ribosomes in eukaryotic cells [36, 59]. Several ribosome‐targeting antibiotics have been reported to exert immunosuppressive effects [60, 61]; however, exactly how these antibiotics impair immune cell function was not well understood. We have previously shown that inhibition of mitochondrial translation using compounds such as argyrin C or linezolid leads to progressive disruption of oxidative phosphorylation (OXPHOS) and reduces effector cytokine production in Teff cells [62]. Because these findings suggested that blocking mitochondrial translation could be a strategy to suppress harmful T helper cell responses, we examined whether the effect of thiostrepton on Foxp3 expression in Tregs could derive from its mitochondrial translation‐inhibitory properties. Initial western blot analysis confirmed that thiostrepton inhibits mitochondrial translation, similarly to the control antibiotic thiamphenicol, as evidenced by decreased COX‐1 expression in Tregs (Figure 5A). We then polarized naïve CD4⁺ T cells under T_H_1‐ or iTreg‐inducing conditions in the presence of DMSO or thiostrepton, and assessed mitochondrial activity through Seahorse assays. Both cell types showed reduced oxygen consumption rates (OCR), with a more pronounced decrease in thiostrepton‐treated Tregs (Figure 5B). In line with these observations, thiostrepton treatment significantly lowered both basal and maximal OCR, as well as ATP production and spare respiratory capacity (SRC) in iTregs (Figure 5C). However, inhibition of mitochondrial translation by thiostrepton alone does not fully explain the impairment in iTreg differentiation, as neither linezolid nor thiamphenicol, two other strong inhibitors of mitochondrial translation [62], led to a decrease in Foxp3 expression (Figure 5D,E). Therefore, mitochondrial dysfunction appears to be a consequence of thiostrepton treatment, but it does not solely account for the failure of iTreg differentiation.

## 7 FOXM1 modulation Does Not Affect Foxp3 Levels in Treg Cells

**FIGURE 6:**
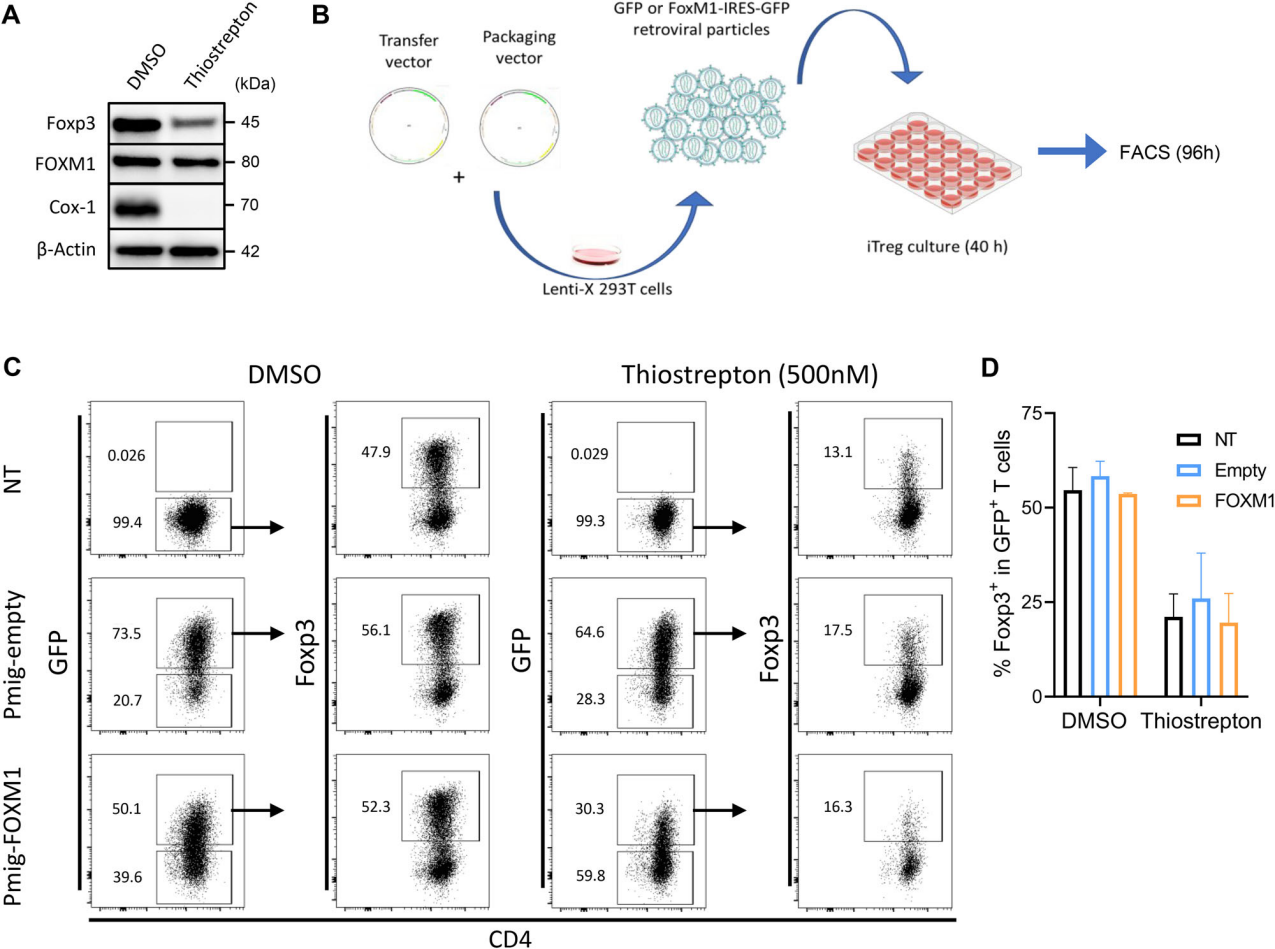
**FOXM1 modulation does not affect Foxp3 levels in Treg cells**. **(A)** Mouse naïve T cells were cultured in iTreg‐polarizing conditions in the presence of DMSO or thiostrepton (500 nM). Foxp3, FOXM1, Cox‐1, and β‐Actin protein levels assessed by western blot. Corresponding raw data in Figure S8B. **(B)** Schematic of experimental design: naïve T cells were cultured under iTreg‐polarizing conditions in the presence of either DMSO or thiostrepton, transduced with retroviruses encoding FOXM1 or GFP‐only (empty) constructs, and further analyzed for flow cytometry. **(C)** Representative plots showing GFP and Foxp3 expression and **(D)** bar graph showing the frequency of Foxp3^+^ cells within the transduced iTregs. Values are expressed as the mean ± SD, and asterisk values are significant compared with the control group by two‐way ANOVA. **(A)** *N* = 3, *n* = 2. **(C, D)** *N* = 3, *n* = 5.

FOXM1, a member of the Forkhead family of transcription factors, is a regulator of cell cycle progression [63, 64]. Several studies have demonstrated that thiostrepton directly suppresses FOXM1's transcriptional activity in vitro, preventing its binding to genomic target sites and breaking its auto‐regulatory feedback [42, 43, 65]. Therefore, we investigated whether FOXM1 might mediate the effect of thiostrepton on Treg cells. To this end, we created FOXM1^flox/flox^ Foxp3^cre‐YFP/Y^ conditional knockout mice, which lack FOXM1 expression specifically in Treg cells. We monitored 16 mice over 97 weeks and failed to observe any evident organismal signs of Treg dysfunction, such as lymphoproliferation and multiorgan inflammation (skin, lungs, liver) (data not shown). Western blot analysis comparing iTreg cells generated in vitro in the presence of DMSO or thiostrepton also confirmed that, within the doses routinely used to inhibit Treg differentiation, FOXM1 expression in Treg cells is not affected (Figure 6A).

We then tested whether overexpressing FOXM1 in Treg cells treated with thiostrepton would restore Foxp3 levels. Thus, iTreg cells were transduced with retroviral vectors encoding FOXM1 or the corresponding empty expression cassette, using IRES‐GFP as a reporter, and analyzed them at day 4 of culture (Figure 6B). The gating strategy is depicted in Figure S8A. Overexpression of FOXM1 failed to restore Foxp3 levels to those of DMSO‐treated cells (Figure 6C,D; Figure S8C–E). Collectively, these data allow us to conclude that FOXM1 is not involved in the mechanism through which thiostrepton impairs Foxp3 expression in Treg cells.

## 8 Thiostrepton Inhibits Human iTreg Differentiation

**FIGURE 7:**
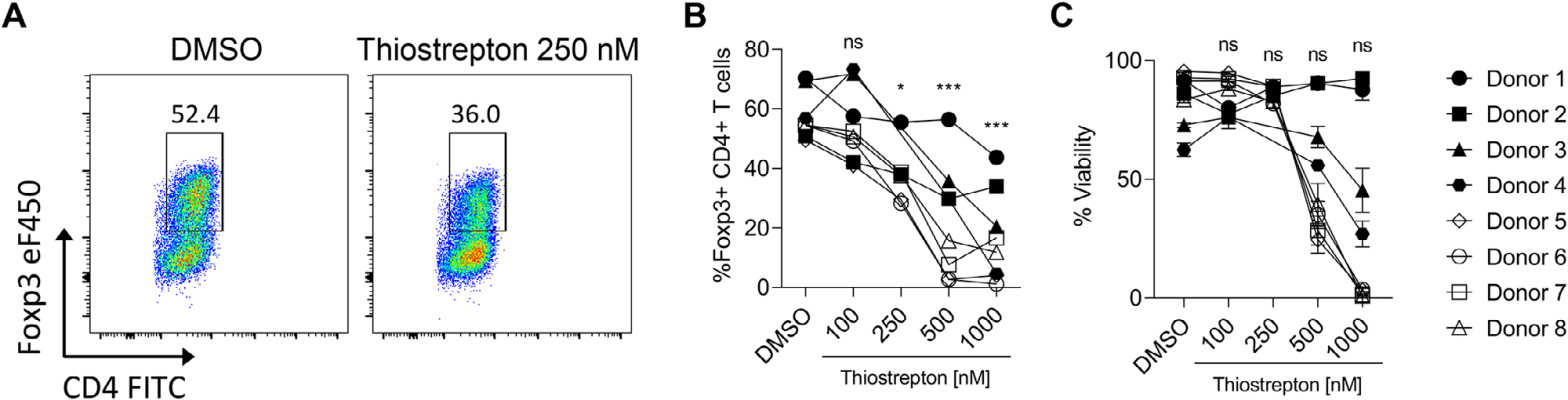
**Thiostrepton inhibits human iTreg differentiation**. Naïve T cells were isolated from the peripheral blood of human donors, polarized under iTreg‐inducing conditions, and cultured in the presence of DMSO or thiostrepton at the indicated concentrations. **(A)** Representative plots, **(B)** frequency of Foxp3^+^ iTregs, and **(C)** viability of the cells. The Multiple unpaired *t*‐test was used for statistical analysis (ns, not significant). **(A–C)** *N* = 8, *n* = 3.

To assess the clinical significance of our findings and determine whether thiostrepton could also inhibit human iTreg differentiation, we isolated naïve CD4 T cells from the peripheral blood of healthy human volunteers and cultured them under iTreg‐polarizing conditions in the presence of DMSO or increasing concentrations of thiostrepton. Consistent with our murine data, 500 nM of thiostrepton significantly reduced the frequency of Foxp3‐expressing cells by nearly 50% compared with controls (Figure 7A). Notably, in most human samples, this decrease was already observed at 250 nM of thiostrepton (Figure 7B), with cell viability only affected at 500 nM–1 µM (Figure 7C). These findings demonstrate that thiostrepton inhibits iTreg differentiation in both murine and human systems, supporting its potential translational relevance as a Treg‐targeting agent in cancer immunotherapy.

## 9 Discussion

Thiostrepton has been reported to exert antitumor activity in vivo [39, 40, 42, 43, 44, 65]. Accordingly, in the present study, our data show that treatment with thiostrepton was sufficient to reduce tumor growth in mice with experimentally‐induced MC38 tumors. This antitumor effect has been mainly attributed to the inhibition of FOXM1 in tumor cells by thiostrepton, which causes cell cycle arrest and impaired proliferation [39, 42, 43, 63, 65]. Strikingly, our experiments revealed a reduced proportion of intratumoral Tregs in thiostrepton‐treated mice, a finding that challenges what was previously described as a sole direct effect on tumor cells. Since Tregs are associated with a poor prognosis and therapy response [66], our study suggests that the hitherto‐reported antitumor effects of thiostrepton may also involve its inhibition of intratumoral Tregs in vivo, and not just a direct effect on the tumor cells.

To date, the effects of thiostrepton on primary murine cells have remained poorly characterized; however, our observations indicate marked cytotoxicity in primary murine T helper cell cultures at concentrations of 2 µM and above (data not shown). Such concentrations have been routinely used in several publications when treating tumor cell lines in vitro to show thiostrepton's antiproliferative effects [40, 42, 44]; however, this outcome is highly variable among the different cell lines, with doses up to 10 µM not significantly affecting cell growth of breast cancer cell lines [39]. Moreover, the direct downregulation of FOXM1 expression is only observed at treatment doses of 5 µM and higher [39, 40]. Our in vitro experiments demonstrated that thiostrepton failed to directly inhibit the growth of MC38 cells at doses up to 1 µM, however, treatment with nanomolar concentrations was already sufficient to inhibit the differentiation of Tregs, compromising not only Foxp3 expression and suppressive function, but also decreasing surface expression of bona‐fide Treg activation and functional markers such as CTLA‐4, CD25, and GITR, without cytotoxic effects. At Treg‐inhibitory doses, early in vitro T cell activation and T_H_1 polarization and function were largely unaffected (data not shown). Therefore, our findings indicate that thiostrepton preferentially targets Tregs over other key players of the TME. This unique property may explain the antitumor effect we observed as a result of thiostrepton treatment and highlights thiostrepton as a potential candidate for addressing the urgent and unmet clinical need for a pharmacological inhibitor of Tregs in vivo as a way to boost antitumor immunity.

Most of the antitumor effects of thiostrepton have been attributed to its direct inhibition of FOXM1 and subsequent arrest in cellular proliferation. Thiostrepton selectively downregulates FOXM1 mRNA expression and activity, resulting in senescence and apoptosis of several types of cancer cells [37, 38]. This transcription factor plays a crucial role in cell cycle progression, particularly during the G1/S and G2/M transitions, and is ubiquitously expressed in highly proliferating cells [67]. Unexpectedly, we found that thiostrepton does not inhibit FOXM1 in Tregs at doses required to suppress Foxp3 expression. This may be due to the fact that FOXM1 is minimally expressed in slow‐cycling cells, and Treg cells display lower proliferation rates when compared with Teff subtypes, a characteristic mostly attributed to their distinct metabolic programming and functional roles [68, 69]. A study by Prots, Iryna et al., showed that FOXM1 expression is downregulated in Tregs, decreasing from early to late maturation stages [70]. Furthermore, mice lacking FOXM1 specifically in Tregs did not present organismal signs of Treg dysfunction (data not shown), and ectopic overexpression of FOXM1 was unable to rescue the lower Foxp3 expression in Tregs caused by thiostrepton. We propose that this discrepancy may be due to differences in dose or cell type. Given that Tregs were inhibited by thiostrepton at nanomolar levels, we conclude that the inhibition of Foxp3 occurs through a pathway unrelated to FOXM1.

Despite not observing an inhibition of FOXM1, we routinely found that thiostrepton exerted a mild antiproliferative effect on Tregs. To our knowledge, no studies have explicitly investigated the induction of cell‐cycle arrest within Tregs to directly assess its impact on Foxp3 expression or stability. Additionally, we compared thiostrepton with other well‐known antibiotics that have similar antiproliferative effects, linezolid and thiamphenicol; however, neither replicated the unique inhibitory effect of thiostrepton on Treg cell differentiation. Moreover, inhibition of Foxp3 expression was similarly impaired in cells that underwent different rounds of proliferation, showing that the two processes are not directly linked and that the antiproliferative effect of thiostrepton occurs in parallel to, rather than upstream of, Foxp3 inhibition.

In a previous publication, we demonstrated that blocking mitochondrial translation with compounds like argyrin C or linezolid causes a gradual disruption of oxidative phosphorylation (OXPHOS) and reduces effector cytokine production in Teff cells [62]. Additionally, thiostrepton impacts mitochondrial function in several ways: it inhibits the mitochondrial ribosome, induces mitochondrial damage, and disrupts mitochondrial membrane potential [59, 71]. Over the past decade, genetic studies have established that mitochondrial electron transport chain (ETC) activity and mitochondrial fission/fusion dynamics are essential for regulating multiple aspects of both conventional and regulatory T cell responses in vivo [58].

Moreover, Foxp3 is responsible for enhanced OXPHOS and fatty acid oxidation in Tregs, including upregulation of ETC proteins and increased respiratory capacity [72]. Our data shows that thiostrepton‐treated Tregs exhibit heavily reduced OCR, with significantly lower basal and maximal oxygen consumption rates, ATP production, and SRC, especially when compared with thiostrepton‐treated T_H_1 cells. Because precise mechanisms linking OXPHOS inhibition to Foxp3 regulation remain unclear, and other antibiotics known to impact mitochondrial function, such as linezolid and thiamphenicol, do not affect Foxp3 expression, whether these effects alone can explain the impairment in iTreg differentiation should be further investigated. Regardless, our data suggests that the effects are most likely related but uncoupled. Since mitochondrial OXPHOS depends on the function of the respiratory chain complex, and tumor‐infiltrating Tregs have diverging metabolic requirements from splenic counterparts [73], we can even speculate that, in our in vivo tumor model, thiostrepton treatment would make tumor‐infiltrating Tregs more prone to losing their suppressive capacity and failing to accumulate in the nutrient‐poor, hypoxic TME.

As the current data do not support a role for a specific factor, such as FOXM1, in mediating the effects of thiostrepton on Tregs, the mechanism remains elusive. Thiostrepton is a known proteasome inhibitor and may broadly perturb protein stability, TCR signaling, or metabolic programs, thereby destabilizing Treg suppressive identity. Another hypothesis is that thiostrepton affects other transcription factor networks critical to Treg identity and function, such as IRF4, BATF, and Helios, which together orchestrate the effector Treg program within the tumor microenvironment. In human tumors, IRF4 drives the differentiation of effector Treg subsets, regulating genes associated with activation and suppressive function, including Helios, ICOS, and CCR8, via cooperation with AP‐1 family members such as BATF and BATF3 [74, 75, 76]. IRF4–BATF3 interactions have also been shown to induce metabolic reprogramming—particularly glycolysis—that can interfere with Foxp3 stability and alter Treg differentiation fate [75]. Thiostrepton could therefore impair effector Treg stability by destabilizing IRF4–BATF complexes or altering their downstream transcriptional programs. Moreover, interference with Helios, a transcriptional partner downstream of IRF4 crucial for Treg stability, is another plausible mechanism, particularly since Helios has been linked to the maintenance of suppressive function in tumor‐infiltrating Tregs [77, 78, 79]. Future studies examining thiostrepton's impact on the proteosome, IRF4, BATF, and Helios expression and Treg glycolytic or oxidative phosphorylation profiles would help clarify its mechanism of action in Tregs.

In summary, we demonstrate, for the first time, the effects of thiostrepton in inhibiting Treg cells differentiation and function. Accordingly, we also show that thiostrepton's effect on Treg cells extends to humans, as human iTregs also display significantly diminished Foxp3 levels without cytotoxicity. In our in vivo tumor model, treatment with thiostrepton significantly impacted regulatory T cell function without observable toxicity. This indirect modulation of the tumor microenvironment may offer a more advantageous therapeutic strategy, especially when used as an adjuvant treatment in combination with established therapies. In the future, it would be valuable to explore the translational potential of thiostrepton and evaluate the compound in various other cancer models to better understand which human tumors are more likely to respond to treatment. Given that thiostrepton is currently FDA‐approved for veterinary use, repurposing it as an adjuvant immunotherapy could represent a low‐risk approach to potentiate antitumor immune responses, ultimately improving clinical outcomes in cancer patients.

### 9.1 Data Limitations and Perspectives

Several limitations of the present study need to be acknowledged. First, some experiments proposed during peer review could not be included due to scientific and practical constraints. For instance, while testing our approach in DEREG mice was proposed, previous studies have consistently shown that Treg depletion alone can induce near‐complete tumor rejection in a variety of models [22, 26]. As such, we do not expect that combining thiostrepton with Treg depletion would provide substantial additional mechanistic insight. The present revision also did not allow for a more comprehensive analysis of effector molecule expression in tumor‐infiltrating Tregs. Such analyses will be essential in future studies to better define how thiostrepton modulates Treg activity within the tumor microenvironment. Likewise, we were unable to validate FOXM1 modulation using the conditional knockout model; this remains an important avenue for future work. Lastly, we did not investigate Th2 polarization, as these cells are generally considered less relevant to antitumor immunity. Nevertheless, a more comprehensive assessment of the broader immune context may help clarify whether thiostrepton influences other noninvestigated immune antitumor pathways.

Finally, the present study does not fully delineate the molecular mechanisms by which thiostrepton influences Foxp3 expression and Treg function. Defining these mechanisms will be critical before thiostrepton can be proposed as a potential adjuvant in cancer immunotherapy.

## 10 Material and Methods

### 10.1 Animal Husbandry and in vivo Experiments

#### 10.1.1 Mice

All mice were bred and maintained under specific pathogen‐free conditions at the animal facility of the Helmholtz Center for Infection Research (Braunschweig, Germany), TWINCORE (Hannover, Germany), Medical Center of Johannes Gutenberg University Mainz (Mainz, Germany), or Ribeirao Preto Medical School (Ribeirao Preto, Brazil).

C57BL/6‐Tg(Foxp3‐DTR/EGFP)23.2Spar (DEREG) mice and B6.PL‐Thy1a/CyJ (Thy1.1) mice were described previously [80, 81]. B6.SJL‐Ptprca Pepcb/BoyJ (CD45.1) mice were obtained from the Jackson Laboratory, and C57BL/6JRj were obtained from Janvier Labs.

#### 10.1.2 MC38 model

Female C57B6/J mice between 8 and 12 weeks of age were anesthetized, shaved on the left flank, and injected subcutaneously with 100 µL sterile PBS containing 4 × 10^5^ MC38 cells. The MC38 tumor growth was monitored visually and measured with a caliper as soon as tumors became palpable. A solution of Lecithin in PBS (12.5 mg/mL) was used to dissolve thiostrepton at a concentration of 3.2 mg/mL. The vehicle control consisted of the same solution but with DMSO. A total of 200 µL of these solutions was administered i.p., every second day from days 3 to 17 postinduction, unless otherwise stated. Animals bearing tumors bigger than 1500 mm^3^ or showing other signs of elevated disease burden according to the scoring system indicated in the animal grant were sacrificed according to the institutional guidelines. All animal experiments were performed according to the federal and regional guidelines of animal welfare of the institutions mentioned above, and all efforts were made to minimize the potential suffering of the mice.

#### 10.1.3 Organ Isolation for the Preparation of Single‐Cell Suspensions

##### 10.1.3.1 Lymph Nodes and Spleen

Mice used for organ collection were euthanized by CO_2_ inhalation followed by cervical dislocation. Spleen and lymph nodes were harvested and kept on ice in RPMI (RPMI 1640 GlutaMAX medium [Gibco]), containing 2% FCS (Biochrom), until further processing. For the preparation of single‐cell suspensions, spleen and LNs were mechanically dissociated by gently pressing them through 100 and 70 µm cell strainers, respectively, followed by flushing with RPMI containing 2% FCS. The cell suspensions were centrifuged at 400*g* for 7 min at 4°C. After discarding the supernatant, splenocytes were subjected to red blood cell (RBC) lysis for 1 min at room temperature (RT), washed, and then centrifuged at 400*g* for 7 min at 4°C. The resulting cell suspensions were then further processed and used for downstream applications.

##### 10.1.3.2 Tumor Isolation and Processing

Tumors were carefully excised from euthanized animals, ensuring complete removal of residual skin and adipose tissue. For tissue digestion, tumors were collected in complete RPMI (RPMI 1640 GlutaMAX medium [Gibco], supplemented with 10% heat‐inactivated FCS [Biochrom] and 500 U penicillin‐streptomycin [PAA laboratories]), containing DNAse I (0.05 mg/mL; AppliChem) and Collagenase D (0.5 mg/mL; 440 Roche). The tumors were enzymatically digested for 40 min using the gentleMACS Dissociator (Miltenyi Biotec), selecting the gentle MACS Program 37C_m_TDK_1 (soft/medium), and then passed through a 100 µm cell strainer and centrifuged. The cell pellet was resuspended in 40% Percoll (GE Healthcare) solution and overlaid onto 80% Percoll solution to create a discontinuous density gradient. The gradient was run at 840*g* and RT for 20 min without breaks. The enriched lymphocyte fraction at the interphase was collected, washed, and used for further downstream applications.

#### 10.1.4 Cell Culture

##### 10.1.4.1 Maintenance of Cell Lines

MC38 cells were cultured in cDMEM supplemented with 1 mg/mL Geneticin (Gibco). Cells were split 1:10 every 3 days.

##### 10.1.4.2 Murine T Cell Enrichment

T cells were isolated from spleen and peripheral lymph nodes using the EasySep Mouse CD4^+^ T Cell Isolation Kit (StemCell Technologies) according to the manufacturer's instructions. To enrich naïve T cells, an anti‐CD25 (eBioscience) and anti‐CD44 (eBioscience) antibody cocktail was used in conjunction with the kit.

##### 10.1.4.3 Murine Naïve T Cell Sorting

Cell suspensions were prepared from spleen and peripheral lymph nodes of mice, pre‐enriched with EasySep Mouse CD4^+^ T Cell Isolation Kit (StemCell Technologies) according to the manufacturer's instructions, and naive T cells were sorted based on the expression of extracellular markers as follows: CD4^+^ CD25^−^ CD62L^+^. Murine nTregs were sorted using reporter DEREG mice [80], based on the expression of GFP and extracellular CD4.

##### 10.1.4.4 Ex Vivo Expansion of Murine nTregs

1 × 10^5^ of sorted cells were cultured in 200 µL of cRPMI, in 96‐well F‐bottom cell culture plates, precoated with 1 µg/mL anti‐CD3e (clone 145‐2C11; Bio X Cell) and 10 µg/mL aCD28 (clone 37.51; Bio X Cell). The medium was supplemented with 500 IU/mL rhIL‐2 (Peprotech). Starting on the second day of culture, the cells were supplemented with rhIL‐2 (Peprotech) every other day, transferred to uncoated 96‐well F‐bottom cell culture plates, and kept at 37°C and 5% CO_2_.

##### 10.1.4.5 Murine iTreg cell cultures

Sorted or enriched naïve T cells were cultured in 200 µL of cRPMI. For iTreg induction, 2.5 × 10^4^ naïve T cells were seeded in 96‐well U‐bottom cell culture plates precoated with 5 µg/mL anti‐CD3e (clone 145‐2C11; Bio X Cell).

The medium was further supplemented with 1 mg/mL aCD28 (clone 37.51; Bio X Cell), 200 IU/mL rhIL‐2 (Peprotech), and 1 ng/mL rhTGF‐β1 (Peprotech). On the second day of culture, the cells were supplemented with rhIL‐2 (Peprotech) and kept for another 2 days at 37°C and 5% CO_2_. Thiostrepton (Merck) was added at the indicated concentrations at the onset of the cultures, unless otherwise specified.

##### 10.1.4.6 Proliferation Assays

Enriched or sorted T cells were washed twice with PBS and labeled with CellTrace Violet (5 µM; ThermoFisher)—at 37°C for 7 min. The remaining unbound dye was absorbed by placing the cells in cRPMI on ice for 5 min. Finally, the cells were centrifuged, washed, and cultured as indicated above.

##### 10.1.4.7 Suppression Assay

The suppressive capacity of nTregs or iTregs was tested by performing an in vitro suppression assay as described previously [82]. For this, we made use of the DEREG mouse model, which allows for precise identification and isolation of Foxp3⁺ Tregs based on the selective expression of GFP under the control of the Foxp3 promoter. Sorted nTregs were incubated with thiostrepton (200 nM) for 24 h prior to the assay, while iTregs were polarized as described previously, in the presence or absence of thiostrepton (200 nM), and sorted at day 4 of culture based on GFP expression.

Naïve T cells from Thy1.1 or CD45.1 transgenic mice were isolated by CD4 enrichment and subsequently labeled with CellTrace Violet dye and seeded at a density of 5 × 10^4^ cells per well in 200 µL of a 96‐well U‐bottom plate, together with 3 × 10^3^ GM‐CSF BMDCs (as described previously [83]) and 4 µg/mL anti‐CD3e. Tregs were then added at the indicated Treg:Teff ratios, and the percentage of proliferating Teff cells was calculated based on CTV intensity (CTV^low^ cells were considered to be actively proliferating). Negative and positive controls were prepared by omitting anti‐CD3e and Tregs or only Tregs, respectively. After 3 to 5 days in culture, cells were harvested and analyzed by flow cytometry.

##### 10.1.4.8 Metabolism Analysis

Naïve CD4^+^ T cells were plated at a density of 1 × 10^5^ or 2.5 × 10^4^ cells per well under T_H_1 or iTreg cell‐polarizing conditions, respectively, with or without thiostrepton, harvested after 96 h of culture, and plated on 96‐well XF cell culture microplates in XF assay medium (pH 7.4, both from Agilent) supplemented with 10 mM D‐glucose, 1 mM sodium‐pyruvate (Merck), and 2 mM L‐glutamine (Gibco). Microplates were incubated for 45 min at 37°C in a non‐CO_2_ incubator and subjected to real‐time analysis of OCR using an XFe96 Extracellular Flux Analyzer (Agilent). For the mitochondrial stress assay analysis, the XF Mitochondrial Stress Test was performed according to the manufacturer's instructions, using subsequent injections of oligomycin (1 µM), FCCP (1.5 µM), and rotenone and antimycin A (0.5 µM).

##### 10.1.4.9 Human Naïve T Cell Isolation from Peripheral Blood

Human peripheral blood from healthy adult volunteers was provided by the blood bank Springe or the Transfusion Medicine Department of the University Medical Center Mainz. To obtain lymphocytes from buffy coats, firstly, the blood was transferred into large sterile flasks and mixed in a 1:2 ratio with warm sterile PBS. Then, 25 mL of this mixture was carefully overlayed onto 15 mL of warm Pancoll solution (PAN Biotech). The tubes were spun at 2000 rpm and RT for 20 min with minimal acceleration and without breaks. The resulting interphase was withdrawn from the gradient and washed twice. After this step, the isolated cells were used immediately for downstream assays or frozen in FCS containing 10% DMSO (AppliChem) at −150°C for later usage. Human CD4^+^ T cell enrichment was performed using the CD4^+^ T cell isolation kit, human (Miltenyi Biotec), according to the manufacturer's instructions. To obtain naïve T cells, the enriched CD4^+^ T cells were sorted based on the expression of extracellular markers as follows: CD3^+^ CD4^+^ CD25^−^ CD127^+^ and CD45RA^+^.

##### 10.1.4.10 Human iTreg Differentiation In Vitro

Sorted human naïve T cells were used to induce iTregs in vitro. After the sort, the cells were rested overnight in cRPMI. The next day, cells were washed, counted, and resuspended in X‐VIVO15 medium (Lonza; BE02‐060Q) at 1 × 10^6^ cells per mL. The cells were then cultured in 96‐well U‐bottom plates, precoated with 5 µg/mL anti‐CD3e Ultra‐LEAF (clone OKT3; BioLegend), in the presence of 100 U/mL rh‐IL‐2 (Peprotech), 5 ng/mL rh‐TGFb1 (Peprotech), 10 nM Retinoic Acid (Sigma‐Aldrich), and 1 µg/mL human CD28 Ultra‐LEAF (clone CD28.2; BioLegend). On day 5 of culture, iTregs were collected and analyzed by flow cytometry.

##### 10.1.4.11 Retrovirus Production and Transduction

The FOXM1 vector was derived from pM2IG (IRES‐GFP) [84] by introducing FOXM1 (2274 bp) into EcoRI and XhoI sites. Retroviral genomes were packaged into retroviruses by co‐transfecting 293T cells with the pM2IG‐FOXM1 vector plasmid and the pCL‐eco packaging plasmid [85], mixed with 1:1 parts jetOPTIMUS reagent (Polyplus) according to the manufacturer's protocol.

Culture medium was changed 12 h after the transfection, and culture supernatant containing retrovirus was harvested after 24 h. Retroviral supernatant collected through 0.45 µm filters was added to proliferating iTreg cells on day 2 of culture, in the presence of 8 µg/mL Polybrene (Santa Cruz Biotechnology) in a 24‐well plate. Plates were then centrifuged at 2000 rpm at 32°C for 1 h to ensure efficient transduction.

##### 10.1.4.12 Real‐Time Quantitative PCR

Total RNA was extracted using the RNeasy Mini Kit (Qiagen), and cDNA was prepared using the SuperScript III reverse transcriptase and random primers (Invitrogen). For real‐time quantitative PCR (RTQ‐PCR), 100 ng cDNA was added to SYBR Green Master Mix (Applied Biosystems) and run in the 7900 HT Fast Real‐time PCR System (Applied Biosystems). The cycling program was 95°C for 20 min followed by 40 cycles of 95°C for 15 s and 60°C for 1 min. Each sample was assayed in triplicate, and the results were normalized to the level of housekeeping gene β‐Actin.

##### 10.1.4.13 Wound‐Healing Assay

Cells were seeded to 80% confluence in a monolayer in 96‐well flat‐bottom plates. A wound was created by firmly scratching with a 2 µL pipette tip. Time‐lapse images were taken by the Cytation 5 cell imaging reader (Agilent BioTek) and analyzed by the Gen5 software (Agilent BioTek).

##### 10.1.4.14 Flow Cytometry

All cells were collected, harvested, and labeled with the LIVE/DEAD Fixable Aqua (ThermoFisher) and Live/Dead Fixable viability dye 780 (Thermo Fisher Scientific) according to the manufacturer's instructions. Afterward, the cells were washed with PBS containing 0.25% bovine serum albumin (BSA), 2 mM EDTA, 0.02% sodium azide (PBA‐E), and endogenous FcRIIy receptors were blocked using self‐made Fc‐Block (murine samples) or human Fc‐Block (BD) on ice. Next, the surface staining was performed on ice by treating the cells with appropriate dilutions of the antibodies of interest diluted in PBA‐E. After the incubation, the samples were washed and fixed with the eBioscience Foxp3/Transcription Factor Staining Buffer Set (eBioscience) for transcription factor staining or 2% PFA for intracellular cytokine staining. After fixation, cells were incubated with appropriate dilutions of the antibodies of interest in PBS containing 0.25% bovine serum albumin (BSA), 0.5% saponin, and 0.02% sodium azide (PBA‐S). Finally, cells were washed, resuspended in PBA‐E, and analyzed by flow cytometry in one of the following cytometers: BD LSR II (BD Biosciences), FACS Symphony (BD Biosciences), Cyan ADP (Beckman Coulter), or CytoFLEX S (Beckman Coulter). The following flow cytometry antibodies were used in this study: murine anti‐CD25 (PC61.5), anti‐CD44 (IM7), anti‐CD8a (53‐6.7), anti‐CD69 (H1.2F3), anti‐CD62L (MEL‐14), anti‐CD4 (GK1.5), anti‐Foxp3 (FJK‐16s), anti‐IFN‐γ (XMG1.2), anti‐TNF‐α (MP6‐XT22), anti‐PD‐1 (J43), anti‐ICOS (7E.17G9), anti‐CTLA‐4 (UC10‐4B9), anti‐GITR (DTA‐1), anti‐Granzyme B (16G6), anti‐CD3e (17A2), anti‐CD45.1 (A20). Human anti‐CD3e (SK7), anti‐CD25 (BC96), anti‐Foxp3 (236A/E7), anti‐CD127 (C262.16A), CD45RA (HI100), anti CD45RO (UCHL1). All murine antibodies are from eBioscience. Cells were analyzed using FlowJo software (Treestar). Experiments were conducted in adherence to the guidelines for the use of flow cytometry and cell sorting in immunological studies.

## Author Contributions

Tim Sparwasser conceived the project. Luana Silva, Fatima Al‐Naimi, Luís Almeida, Daniele Carvalho Nascimento, and Aleksandra Lopez Krol performed the investigations. Luis Eduardo Alves Damasceno, Hakim Echchannaoui, Luciana Berod, and José Carlos Alves‐Filho provided critical discussion. Luana Silva, Fatima Al‐Naimi, Luís Almeida, Luciana Berod, and Tim Sparwasser contributed to manuscript writing and project visualization. Tim Sparwasser supervised the work and handled project administration. Tim Sparwasser and Luciana Berod acquired funding.

## Conflicts of Interest

The authors declare no conflicts of interest.

## Ethics Statement

All experiments involving mice were conducted in accordance with the institutional guidelines of the Lower Saxony State Office for Consumer Protection and Food Safety (Niedersächsisches Landesamt für Verbraucherschutz und Lebensmittelsicherheit; LAVES) under the file symbols 17/2597 and 12/0724, by Fatima Al‐Naimi; as well as the national investigations office Rhineland‐Palatinate (Landesuntersuchungsamt Rheinland‐Pfalz; LUA), under the numbers G‐19‐1‐059 and G 23‐1‐064, by Luís Almeida and Luana Silva, and the Ethics Committee on Animal Use guidelines of Ribeirao Preto Medical School, protocol number 1400/2024, by Luana Silva. Efforts were made to minimize animal suffering.

## Peer Review

The peer review history for this article is available at https://publons.com/publon/10.1002/eji.70035.

## Supporting information




**Supporting File 1**: eji70035‐sup‐0001‐SuppMat.pdf

## Data Availability

The data that support the findings of this study are available from the corresponding author upon reasonable request.
