# Supplementary material for: The Streptomyces Metabolite Thiostrepton Inhibits Regulatory T Cell Differentiation and Function to Boost Antitumor Immune Responses
Source: Eur J Immunol. 2025 Aug 17;55(8):e70035. doi: 10.1002/eji.70035 (PMC12358713; doi:10.1002/eji.70035)
Supplement: Supplementary file 1 — Supporting File 1: eji70035‐sup‐0001‐SuppMat.pdf [file EJI-55-e70035-s001.pdf]

SUPPORTING INFORMATION

The Streptomyces metabolite thiostrepton inhibits regulatory T cell differentiation and function to boost antitumor immune responses

Luana Silva[1*], Luís Almeida[1*], Fatima Al-Naimi[1*], Daniele Carvalho Nascimento[1], Aleksandra Lopez Krol[1], Luis Eduardo Alves Damasceno[1,2], Hakim Echchannaoui[3,4], José Carlos Alves-Filho[1,2], Luciana Berod[4,5] and Tim Sparwasser[1,4]

[1]Institute of Medical Microbiology and Hygiene, University Medical Center of the Johannes Gutenberg-University, 55131 Mainz, Germany

[2]Department of Pharmacology, Ribeirao Preto Medical School, University of Sao Paulo, Ribeirao Preto, SP 14049-900, Brazil; Center for Research in Inflammatory Diseases, Ribeirao Preto Medical School, University of Sao Paulo, Ribeirao Preto, SP 14049-900, Brazil

[3]Department of Hematology, Oncology, and Pneumology, University Medical Center (UMC) and University Cancer Center (UCT), Johannes Gutenberg University, Langenbeckstrasse 1, 55131 Mainz, Germany

[4]Research Center for Immunotherapy (FZI), University Medical Center of the Johannes Gutenberg University Mainz, 55131 Mainz, Germany

[5]Institute for Molecular Medicine, University Medical Center of the Johannes Gutenberg-University, 55131 Mainz, Germany

[*]These authors contributed equally to this work.

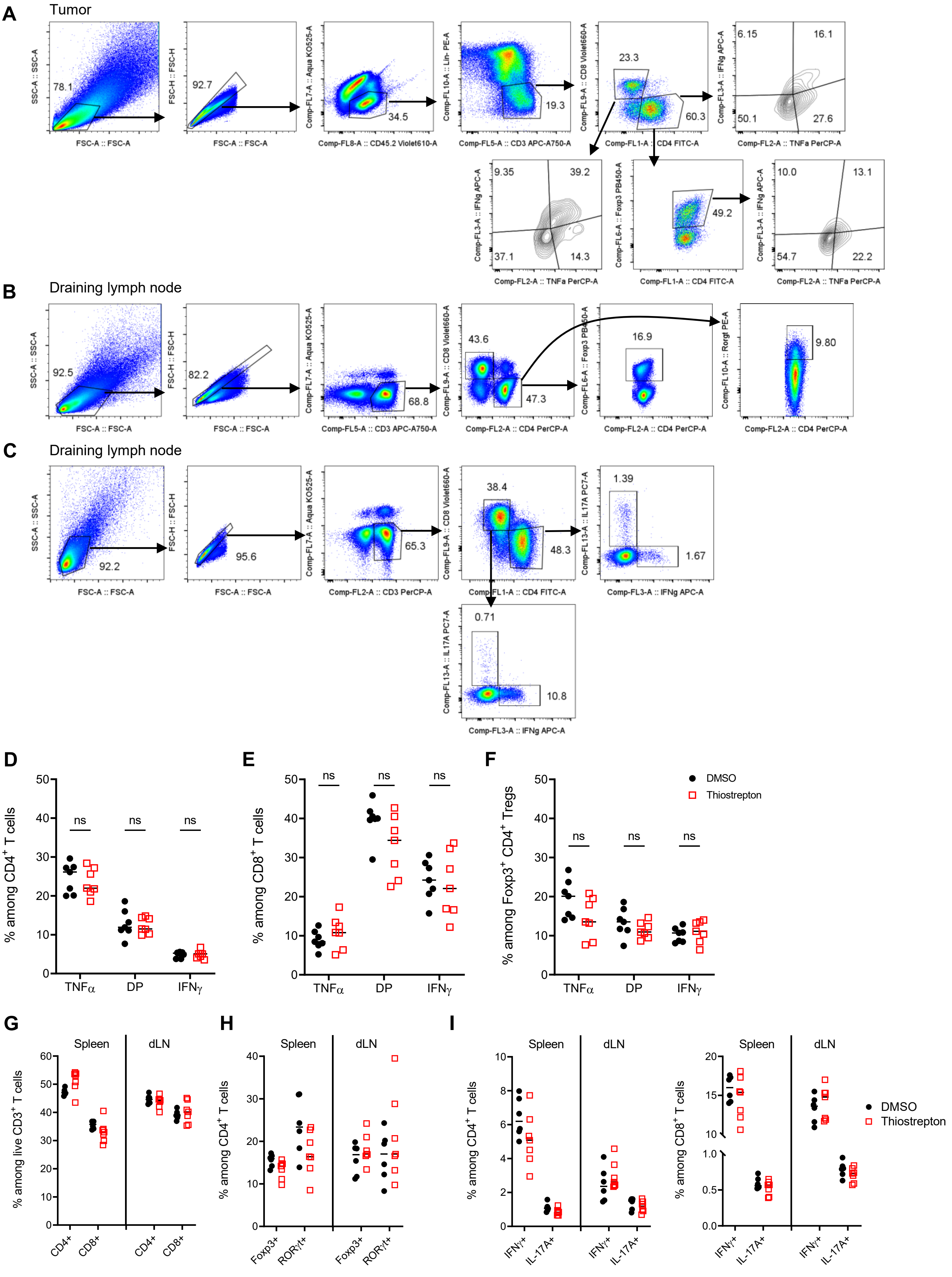
A
Tumor
B
Draining lymph node
C
Draining lymph node
D
% among CD4+ T cells
ns
TNFα
DP
IFNγ
E
% among CD8+ T cells
F
% among Foxp3+ CD4+ Tregs
DMSO
Thiostrepton
G
% among live CD3+ T cells
Spleen
dLN
CD4+
CD8+
H
% among CD4+ T cells
Foxp3+
RORγt+
I
IFNγ+
IL-17A+
% among CD8+ T cells

Supplementary Figure 1: Inflammatory responses of $CD4^+$ and $CD8^+$ T cells in MC38 tumor model upon thiostrepton treatment

(A) To analyze the cellular composition and cytokine production within tumor infiltrating lymph nodes, in CD4, CD8 and Treg populations, cells were gated as depicted in the FACS plots. **(B)** Representative gating strategy of cellular composition within the draining lymph nodes (same analysis applies to the spleen). **(C)** Representative gating strategy of cytokine production within draining lymph nodes (same analysis applies to the spleen). Frequency of TNF-α^+, TNF-α^+ IFN-γ^+ (double positive, DP), and IFN-γ^+ cells within **(D)** $CD4^+$ T cells; **(E)** $CD8^+$ T cells and **(F)** $CD4^+$ $Foxp3^+$ Treg cells isolated from the tumor fraction. **(G)** Frequencies of $CD4^+$ and $CD8^+$ T cells among live cells of the spleen and draining lymph nodes (LN). **(H)** Frequencies of Tregs and RORγ^+ cells among $CD4^+$ cells of the spleen and lymph nodes. **(I)** Frequency of IFN-γ^+ and IL-$17A^+$ cells within $CD4^+$ T cells (right) and $CD8^+$ T cells (left). The experiment was performed using N=7 mice per treatment group (DMSO/Thiostrepton). The Multiple unpaired t test was used for statistical analysis. Mean ± SD, * $p < 0.05$, ** $p < 0.01$, *** $p < 0.001$, ns, not significant.

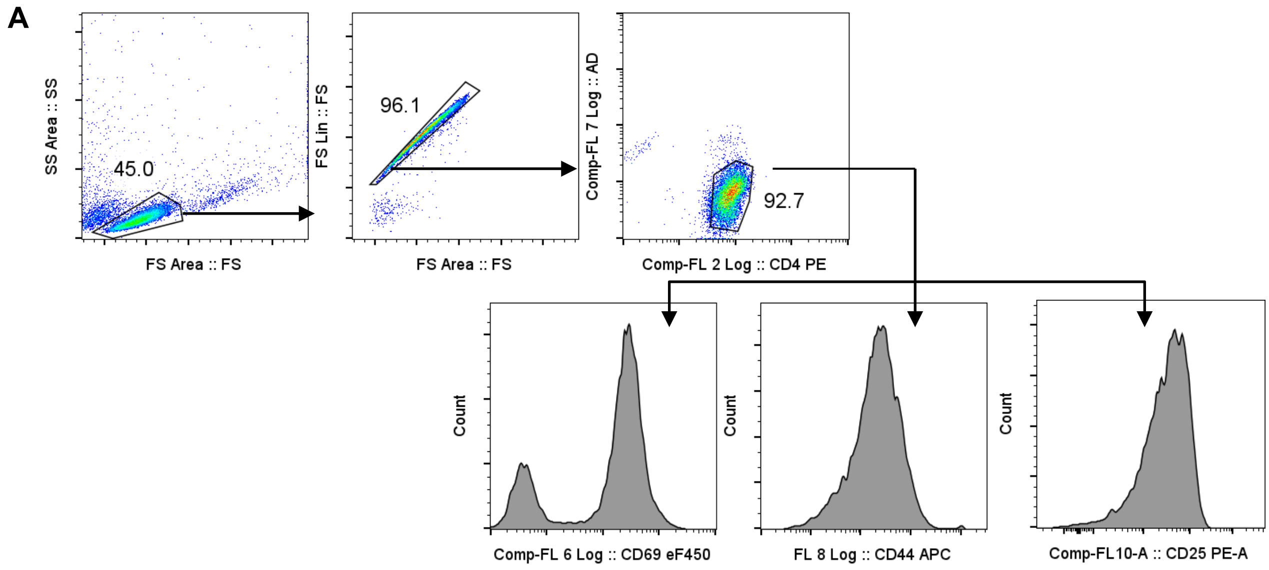

Supplementary Figure 2: Gating strategy from flow cytometry analyses of early T cell activation.

(A) To analyze the T cell activation (CD69, CD44 and CD25 expression), cells were gated as depicted in the FACS plots.

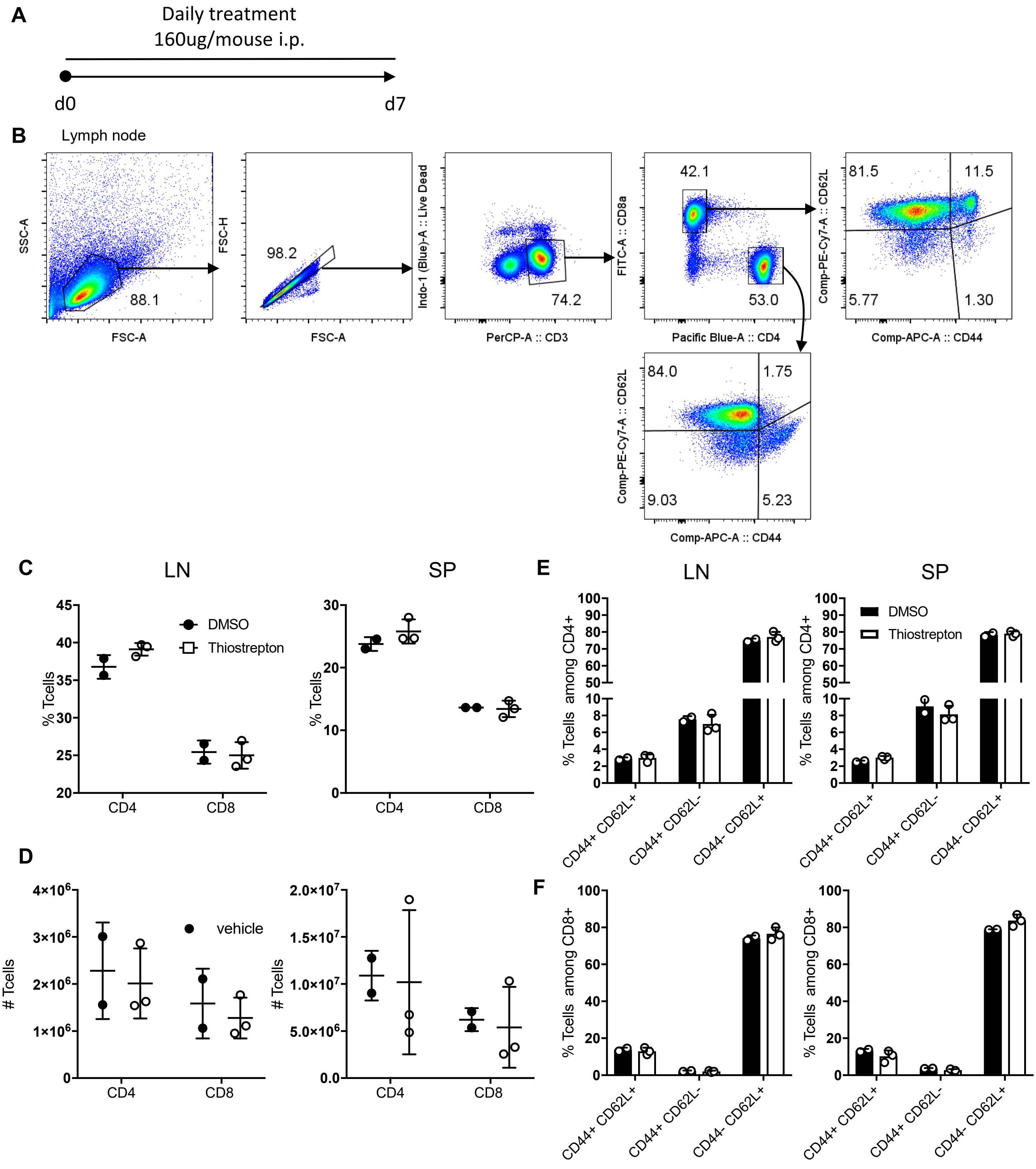

Supplementary Figure 3: Thiostrepton treatment does not influence T cells under homeostatic conditions. (A) Schematic overview. **(B)** Representative gating strategy depicting cellular composition and activation status of cells within lymph nodes of vehicle or thiostrepton treated mice (same analysis applies to the spleen). **(C-F)** Lymphocytes from the lymph nodes (left) and spleen (right) of vehicle or thiostrepton treated mice were isolated and analyzed by flow cytometry. CD4 and CD8 frequencies **(C)** and absolute numbers **(D)** within live cells. Activation status of CD4 **(E)** and CD8 **(F)** T cells. Values are expressed as the mean ± SD. **(A-F)** N=2-3, n= 3.

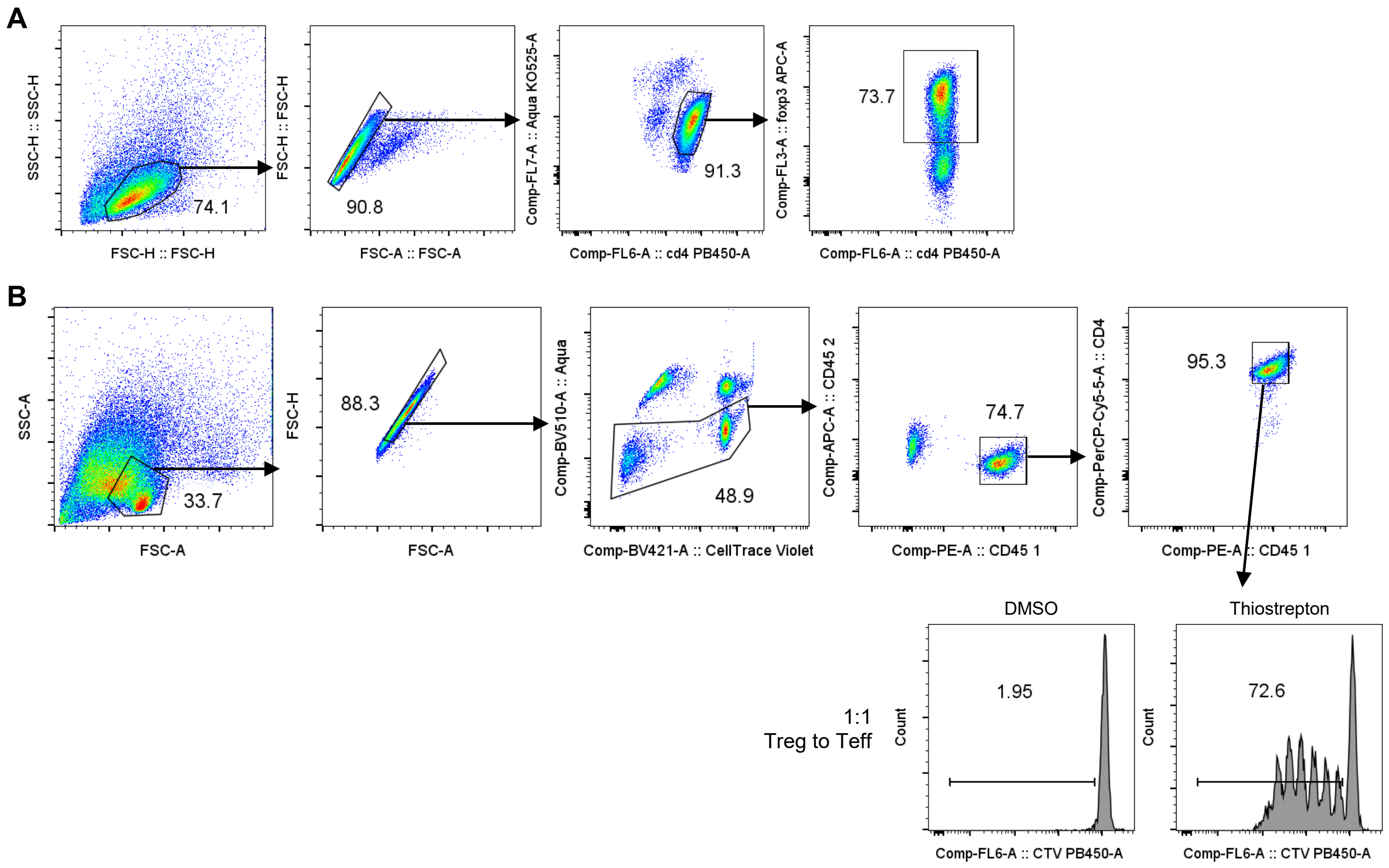

Supplementary Figure 4: Gating strategy from flow cytometry analyses of Treg cell differentiation and function. (A) To analyze the frequency of Foxp3^{+} iTregs generated from polarizing naïve T cells polarized under iTreg inducing conditions in the presence of DMSO or thiostrepton, cells were gated as depicted in the FACS plots. **(B)** Representative gating strategy of the deriving results from in vitro Treg suppression assays.

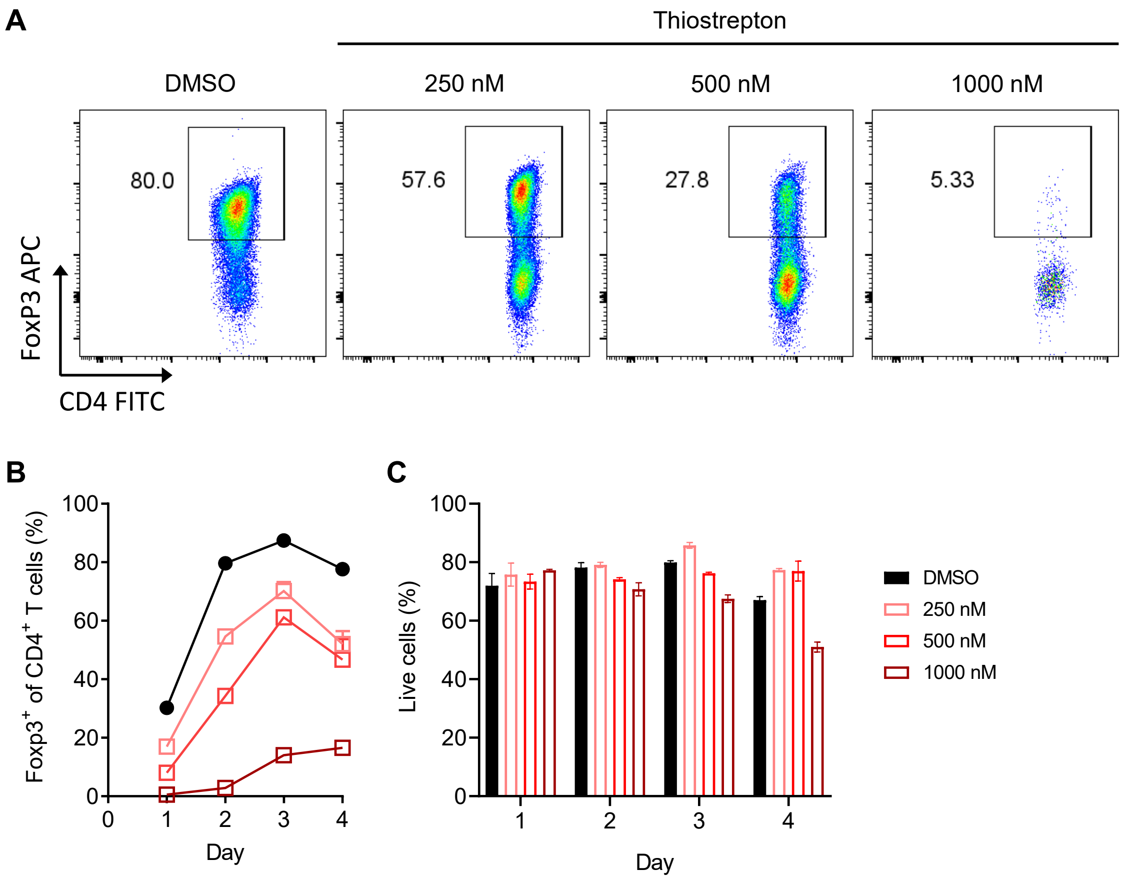

Supplementary Figure 5: Time-dependency of thiostrepton inhibition of iTreg differentiation.

Naïve T cells polarized under iTreg inducing conditions were cultured in the presence of DMSO or thiostrepton at the indicated concentrations. **(A)** Representative contour plots corresponding to Foxp3 percentages at day 2 **(B)** Frequency of Foxp3⁺ T cells in iTreg cultures over time. **(C)** Frequency of viable cells in the presence of vehicle or thiostrepton. Values are expressed as the mean ± SD. **(A-C)** N=2, n=3.

A

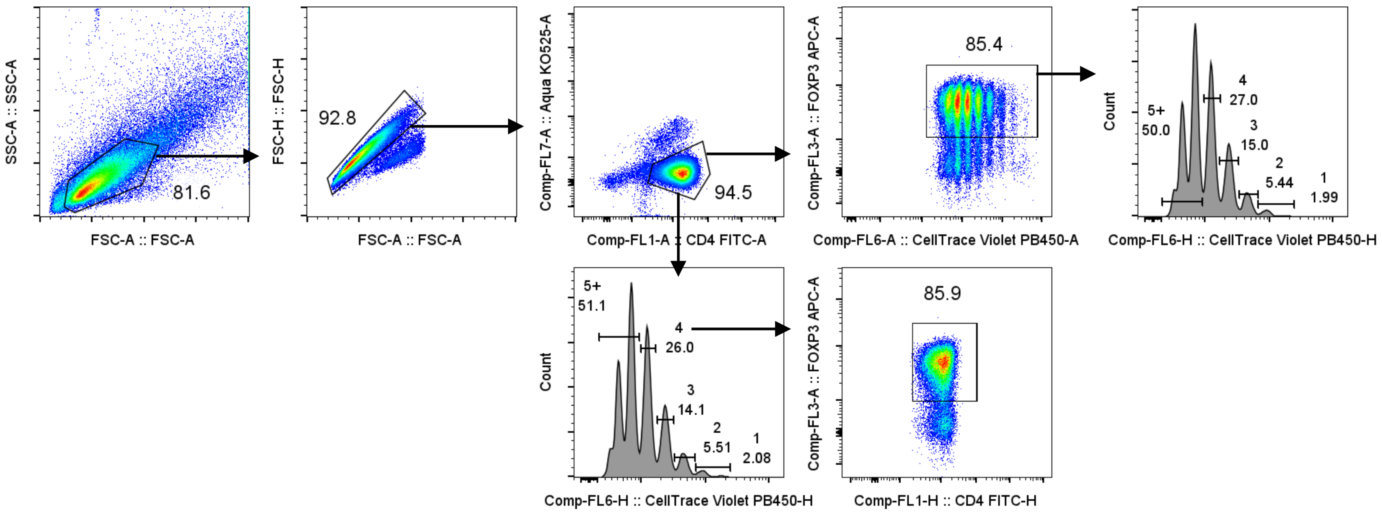

Supplementary Figure 6: Gating strategy from flow cytometry analyses of cellular proliferation experiments.

(A) Representative FACS gating strategy to analyze different proliferation cycles of iTregs and Foxp3 frequencies across each proliferation cycle of CD4^{+} cells, in the presence of DMSO or thiostrepton.

A

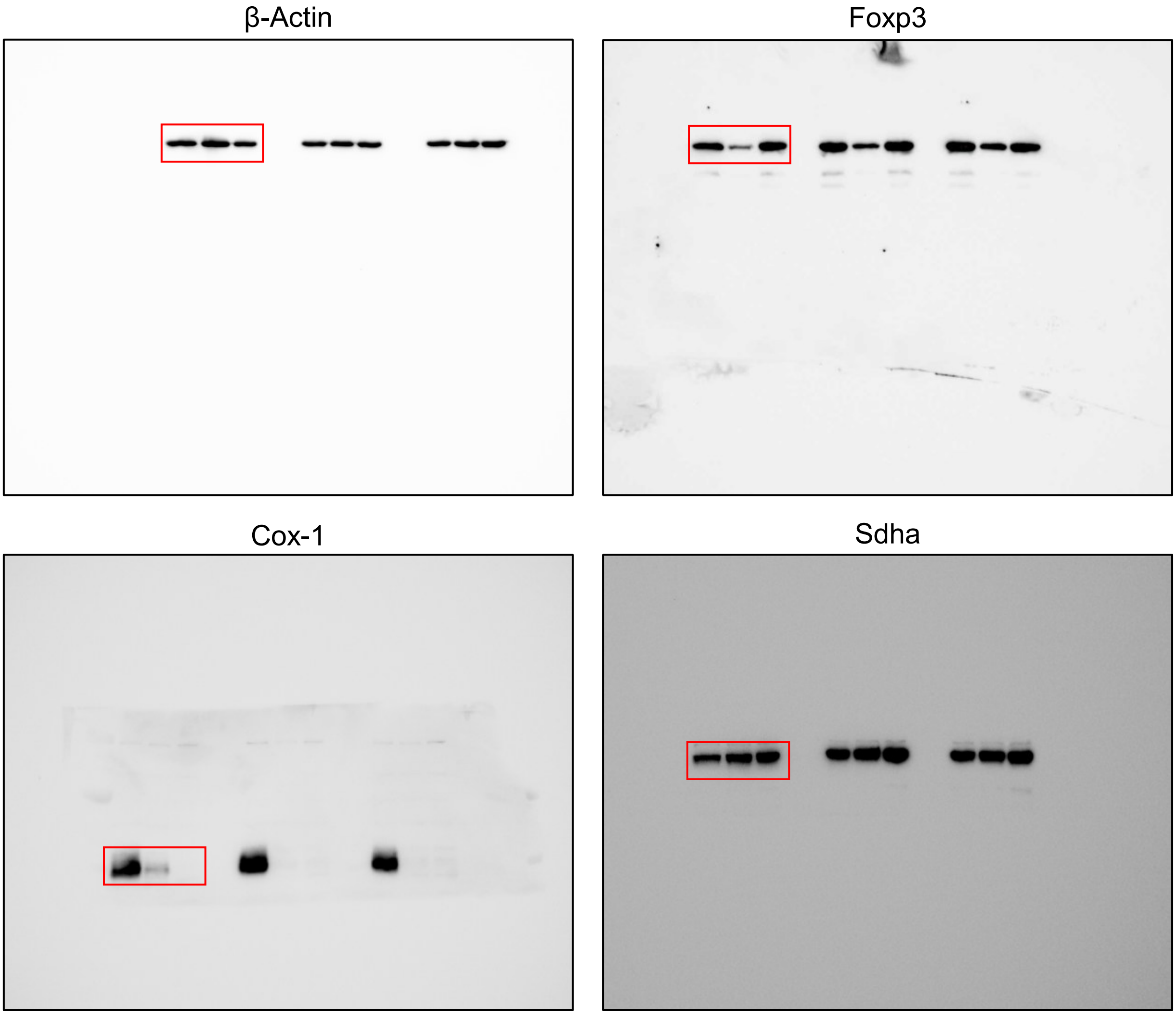

Supplementary Figure 7: Western Blot raw data pertaining to Figure 5

(A) Sections highlighted in red correspond to cuts shown in Figure 5A.

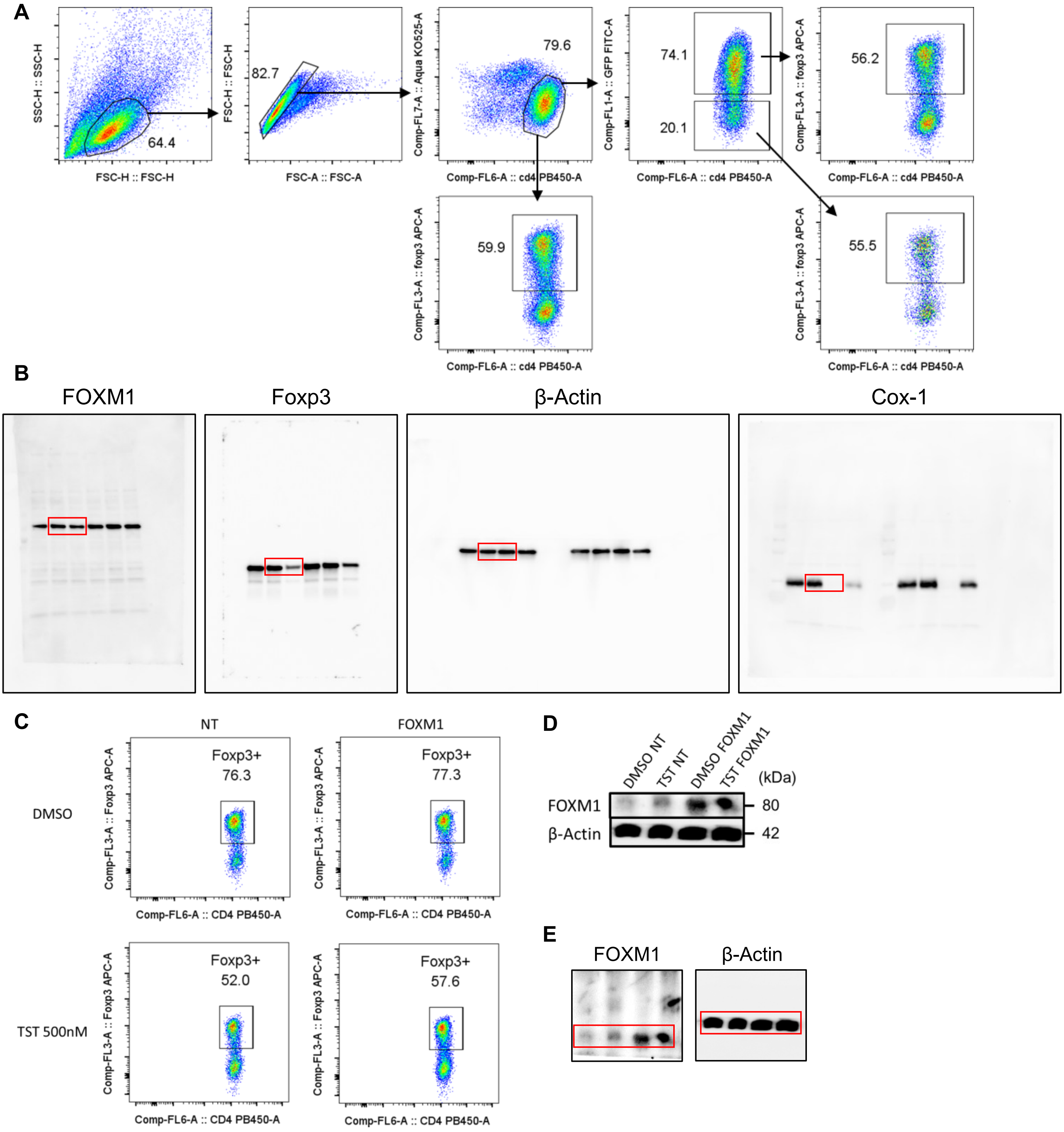

Supplementary Figure 8: Raw data and validation of analyses of FOXM1 overexpression.

(A) To analyze naïve T cells cultured under iTreg-polarizing conditions and transduced with retroviruses encoding FOXM1 or GFP-only (empty) constructs, in the presence of either DMSO or thiostrepton, cells were gated as depicted in the FACS plots. **(B)** Western blot raw data, sections highlighted in red correspond to cuts shown in Figure 6A. **(C)** Validation of Foxp3 levels within CD4+ cells transduced with retroviruses encoding FOXM1 constructs, in the presence of either DMSO or thiostrepton. **(D)** Western blot analysis to validate FOXM1 overexpression in our in vitro system. **(E)** Western blot raw data pertaining to (D) with sections shown highlighted in red.
